# Global Prevalence of Sleep-Disordered Breathing in Intracerebral Hemorrhage Survivors: A Meta-Analysis and Systematic Review

**DOI:** 10.3390/neurolint18010019

**Published:** 2026-01-20

**Authors:** Farhan Ishaq

**Affiliations:** 1 Center for Connected Care, Innovation & Implementation Research, Houston Methodist Academic Institute, Houston Methodist Hospital, Houston, TX 77030, USA; fishaq@houstonmethodist.org; 2 Houston Methodist Research Institute, Houston Methodist Hospital, Houston, TX 77030, USA

**Keywords:** AHI: apnea–hypopnea index, OSA: obstructive sleep apnea, ICH: intracerebral hemorrhage, POSA: positional obstructive sleep apnea, SDB: sleep-disordered breathing

## Abstract

Background: Sleep-disordered breathing (SDB) and intracerebral hemorrhage (ICH) share a bidirectional relationship: SDB may increase ICH risk, while ICH can induce or exacerbate SDB. However, the prevalence and characteristics of post-ICH SDB remain poorly defined. Objective: To estimate the prevalence of SDB among ICH survivors and examine associated clinical factors, including the relative burden of obstructive (OSA) versus central sleep apnea (CSA). Methods: A systematic review and meta-analysis were performed across PubMed, Scopus, CINAHL, and ClinicalTrials.gov. Studies assessing SDB in adults with ICH using American Academy of Sleep Medicine (AASM) category 1–4 diagnostic devices were included. Random-effects models estimated pooled prevalence at varying apnea–hypopnea index (AHI) thresholds, with subgroup analyses by setting, timing, geography, and diagnostic factors. Results: Seventeen studies met inclusion criteria. Pooled SDB prevalence was 85% (95% CI: 80–91%) at AHI > 5, with 49% (95% CI: 42–57%) experiencing moderate SDB (AHI > 15), and 21% (95% CI: 15–27%) experiencing severe SDB (AHI > 30). The prevalence of OSA predominated 73% (95% CI: 64% to 82%),while CSA occurred in 5% (95% CI: 2–9%), corresponding to a pooled RR of 7.44 and OR of 53.08 for OSA versus CSA. Conclusions: SDB—primarily OSA—is highly prevalent following ICH, underscoring the need for early, routine screening and intervention to improve neurological and cardiovascular outcomes.

## 1. Introduction

Stroke is a serious public health problem and one of the leading causes of death among older adults. One type of stroke, intracerebral hemorrhage (ICH), defined as bleeding within the brain parenchyma, represents approximately 10% to 15% of all strokes [1,2]. Although less common than ischemic stroke, ICH is more devastating and has a higher mortality rate; ICH with intraventricular involvement of the brain has demonstrated increased mortality [3]. In the United States, the one-year mortality ratio for ICH is approximately 40% to 65% [4], and the 30-day mortality is estimated to be 35% to 59% [5]. Only 20% of survivors are expected to have full functional recovery at 6 months; half of this mortality occurs within the first 24–48 h [2].

Risk factors for ICH include hypertension, diabetes mellitus (DM), cigarette smoking, excessive alcohol consumption, and trauma [3]. Hypertension and DM cause damage to cerebral blood vessels and can impair healing [6,7]. Cigarette smoking can cause vascular dysfunction [8]. Additionally anatomical abnormalities, impaired healing or weakness of the cerebral blood vessels can cause or contribute to ICH.

Sleep-disordered breathing (SDB) is an umbrella term for sleep-related breathing disorders and are characterized by an abnormal respiratory pattern during sleep. These disorders can cause lack of oxygen and contribute to several negative long-term outcomes. Obstructive sleep apnea (OSA) is the most commonly seen form of SDB; central sleep apnea (CSA) is less commonly seen. OSA is more prevalent in patients who are obese, males, and smokers [9]. It is characterized by either complete or partial obstruction of the upper airway without cessation of respiratory effort during sleep [10]. Obstructive apneas cause sympathetic stimulation and oxidative stress, which disrupts sleep, increases insulin resistance, and causes hypertension. Over time, this may result in many negative outcomes such as impaired healing of the blood vessels, arterial stiffness, and diabetes mellitus [11]. Impaired sleep results in cognitive impairment, psychiatric disorders (depression, anxiety, and bipolar disorder), motor vehicle accidents, workplace accidents, and decreased productivity [12].

The relationship between SDB and ICH is bidirectional. ICH can cause or exacerbate preexisting sleep apnea [13,14]; similarly, one of the risk factors of ICH is sleep apnea, especially OSA [7,15]. There are many complex and synergistic mechanisms involved in the relationship between SDB and ICH [1]. OSA causes vascular dysfunction, including endothelial dysfunction, which can cause both ischemic stroke or ICH. This endothelial dysfunction can cause carotid artery stenosis [16]. CSA is characterized by lack of respiratory effort, resulting in oxygen desaturation, and occurs predominantly in Cheyne–Stokes breathing, a breathing pattern that is commonly seen in CSA [17]. Cheyne–Stokes breathing pattern demonstrates a gradual increase in breathing followed by a decrease and a temporary cessation in breathing [18]. Research has found that CSA may occur more commonly as a result of ICH, especially when infratentorial, and that OSA more commonly precedes ICH [19]. Other studies show that CSA may be transient in ICH and resolves or improves with time [20]. Some studies have suggested that silent cerebral infarcts may cause CSA [21,22]. Carotid artery stenosis may result in baroreceptor and autonomic dysfunction and can result in CSA [23]. The mechanism of mixed sleep apnea, which is the presence of both OSA and CSA with ICH, is poorly understood; more conclusive data is necessary.

The gold standard in diagnosing sleep apnea is an in-lab polysomnography (PSG), which necessitates a technician for the administration and monitoring of the study and can be costly [24]. As costs and availability of staff hinder PSG testing, other more convenient methods can be employed to diagnose sleep apnea. Limited channel devices, also referred to as limited channel polysomnography, are devices that do not use as many channels as a polysomnography, nor do they require a technician to be present for the test. Channels are inputs to the device and can consist of an electroencephalography (EEG) to measure brain activity, electrocardiography (EKG) to monitor the heart, electrooculography to monitor eye movements (EOG), electromyography (EMG) to monitor muscle activity, and pulse oximetry to measure peripheral arterial saturation, as well as two belts each to measure thoracic and abdominal movement and thus breathing. Limited Channel devices are more convenient and less expensive to use. Although they are not as accurate as PSG, they are validated by the American Association of Sleep Medicine for diagnosis [25]. Limited channel devices fall into AASM categories three and four and can have 3–7 channels. Investigation of ICH survivors tolerability of PSG and limited device studies are hindered due to the critical state of ICH patients [26]. Alternative, less disruptive methods of SDB diagnosis in ICH survivors are lacking and not used in routine care [27,28].

There are several factors that affect the presentation and prognosis of ICH and SDB. ICH can vary by topography, size, location, initial insult, perihemotal edema, and regression; SDB can vary with phenotypes such as baroreceptor sensitivity and oropharyngeal anatomy [11,26,29]. This makes it even more difficult to predict who will develop SDB and thus prioritize screening and treatment. Many factors and conditions are common in both SDB and ICH.

Screening for sleep apnea is rare after ICH due to the severity and critical state caused by ICH (high risk of mortality) [30]. Therefore, ICH patients are often excluded from studies that evaluate the relationship between sleep apnea and stroke. As a result, additional research on the relationship between sleep apnea and ICH is needed to develop appropriate prevention and treatment plans for at-risk populations. A defined protocol that increases pragmaticism of screening, including less-invasive screening methods that patients can tolerate, are needed for diagnosis and treatment.

## 2. Objective

To examine and provide an estimate of the prevalence of SDB in ICH survivors. Exploring the prevalence of and associated factors of SDB may help us understand the need for screening and treatment among ICH survivors.

## 3. Methods

### 3.1. Search Strategy

**Figure 1:**
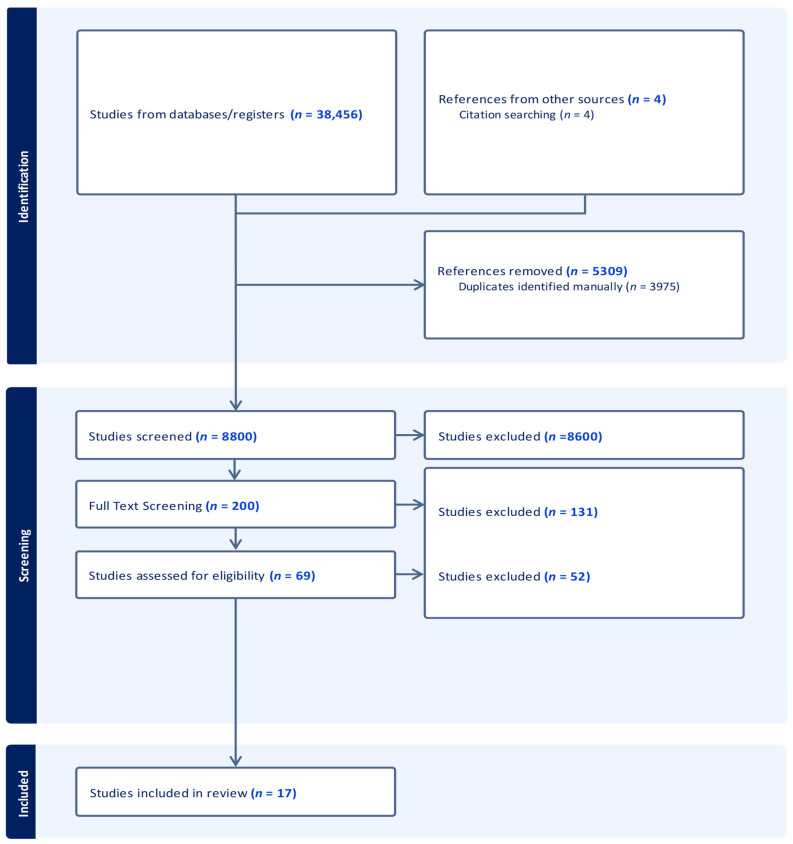
Prisma flow chart.

This systematic review and meta-analysis were conducted in accordance with the PRISMA 2020 reporting guidelines (Figure 1; Supplementary Table S1). The review protocol was registered (Registration DOI: 10.17605/OSF.IO/48GJA). A search was performed with PubMed Scopus, CINAHL, Scopus, and Clinicaltrails.gov using the terms apnea, apnoea intracerebral, hemorrhage, hemorrhage, sleep, disordered, breathing, sleep apnea screening, and setting (Figure 1; Supplementary Materials S1); detailed reasons for study exclusion at both the title/abstract screening and full-text eligibility stages are summarized in Supplementary Materials S2. Additional articles were found by reviewing bibliographies of these studies. SDB was defined as OSA or CSA. Studies were analyzed for sleep study device category in accordance with the American Association of Sleep Medicine (AASM) classification [31,32]. The apnea–hypopnea index (AHI) refers to the number of apneas and hypopneas per hour of sleep for full PSG and per hour of study duration for limited channel studies in which sleep stage was not determined. The respiratory distress index (RDI) is similar to AHI but evaluates respiratory effort-related arousals and also considers respiratory effort-related arousal (RERAS) [31,32]. RERAs are arousals from sleep that do not meet the criteria of apnea or hypopnea events but disrupt sleep. REI refers to Respiratory Event Index and divides event counts by total recording time (vs. sleep time) [31,33]. ODI refers to the oxygen desaturation index [32,34]. Hypopnea definitions were categorized in accordance with the AASM categories of ≥3–4% oxygen desaturation [31,33] and by the device mechanism (hypopnea type). The search included all languages, locations, and years of publication. Studies were limited to adults, defined as 18 years of age or older. When averages could not be obtained, they were estimated from the median [35,36]. The review protocol was not prospectively registered.

### 3.2. Data Extraction

Search results were screened in Mendeley 1.19.5 (Mendeley Ltd., London, UK) [37]. Title/abstract screening and full-text eligibility assessment were conducted by a single reviewer; therefore, no inter-reviewer discrepancy resolution process was required. Abstracts were excluded. Studies were excluded if duplicative data could not be ruled out; if snoring or questionnaire-based methods were used to define sleep apnea; if the prevalence of sleep-disordered breathing (SDB) was not evaluated using an American Academy of Sleep Medicine (AASM) category 1–4 device; or if stratification between ischemic stroke and intracerebral hemorrhage (ICH) was not conducted or could not be deduced. Reasons for full-text exclusion were prospectively defined and are summarized in Supplementary Materials S2.

Data extracted included study characteristics, number of patients with SDB, mean age, mean body mass index (BMI), and sleep-related respiratory indices, including the apnea–hypopnea index (AHI), respiratory disturbance index (RDI), and respiratory event index (REI) (Table 1 and Table 2). Subgroup analyses were performed across established AHI severity thresholds (>5, >10, >15, >20, >30, and >40). These subgroup analyses included patient location (cohort, home, inpatient, rehabilitation unit, and sleep laboratory); timing of assessment after ICH; AASM device category (1–4); full-channel versus limited-channel diagnostic modality; hypopnea criteria (defined as A for 3% desaturation and B for 4% desaturation); hypopnea type; continent; country; timing from ICH to SDB testing; and study design (prospective vs. retrospective) (Supplementary Materials S3).

To accommodate heterogeneity in reported assessment timing and recognized distinctions between acute, subacute, and chronic phases of intracerebral hemorrhage—each associated with differing pathophysiologic processes—two complementary temporal frameworks were used: a coarse framework (<30 days, 30 days–3 months, and >3 months) and a finer acute-phase framework (<7 days, 7–30 days, and >30 days). Results from both frameworks are reported in parallel in subgroup analyses and summarized in Supplementary Materials S3.

Additional clinical and mechanistic variables related to SDB in ICH were extracted when reported from systematically identified studies that met the inclusion criteria. These variables were synthesized descriptively and are summarized in Table 3.

The ROBINS-I tool was used to assess risk of bias across included studies, given their exposure–outcome framework (ICH exposure with subsequent SDB assessment). Although ROBINS-I was originally developed for non-randomized intervention studies, it is widely applied to exposure-based observational research in which confounding, selection, and measurement biases are relevant (Supplementary Materials S4).

### 3.3. Statistical Analysis

A quantitative synthesis of the data from the selected studies was conducted by using random-effects (RE) meta-analyses to account for heterogeneity. Additionally, several parameters were utilized to assess SDB index and positive diagnosis. Subgroup analyses were performed using RE for multiple factors, including patient location, region, country, timing of study after ICH, study type, testing method, hypopnea criteria, hypopnea type, AASM device classification, and hypopnea type. RE meta-analysis of hypertension using meta-regression was performed. All analyses were conducted using Stata 19 (StataCorp, College Station, TX, USA) [51].

## 4. Results

The search identified 8800 possible publications with 3975 duplicates removed. A total of 4254 abstracts were screened in Mendeley, identifying 120 possible publications which were fully screened. Studies were excluded if the prevalence of ICH in SDB was not available or determinable; were abstracts; did not stratify between ischemic stroke or ICH; or used questionnaires, snoring, or non-AASM category 1–4 device as criteria to diagnose SDB (Supplementary Materials S2). A total of 17 publications were included in the meta-analysis (Figure 1; Supplementary Materials S2). Of these seventeen studies, the mean age and BMI were found in five studies and the prevalence of hypertension was found in four studies.

### 4.1. Prevalence of SDB in ICH Survivors

#### Prevalence of Sleep-Disordered Breathing Across AHI Thresholds

A meta-analysis was performed to estimate the prevalence of sleep-disordered breathing (SDB) at varying thresholds of the apnea–hypopnea index (AHI) among patients surviving intracerebral hemorrhage (ICH). The proportion of patients meeting each AHI criterion is summarized in Table 4.

At the lowest threshold (AHI > 5 events/h), pooled data from 10 studies (361 of 419 patients) demonstrated a high prevalence of SDB at 85% (95% CI: 80% to 91%), with moderate heterogeneity (*I*^2^ = 47.5%, *p* = 0.05).

**Figure 2:**
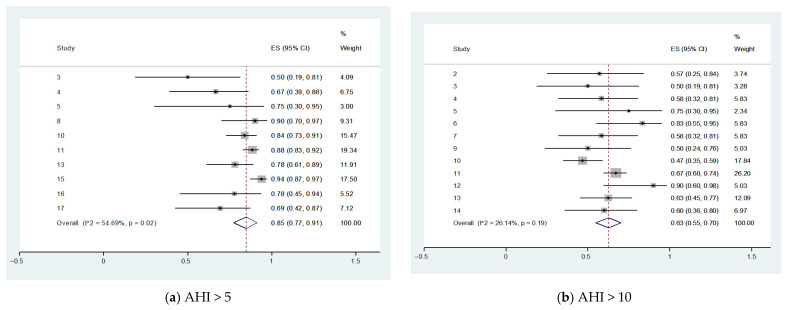

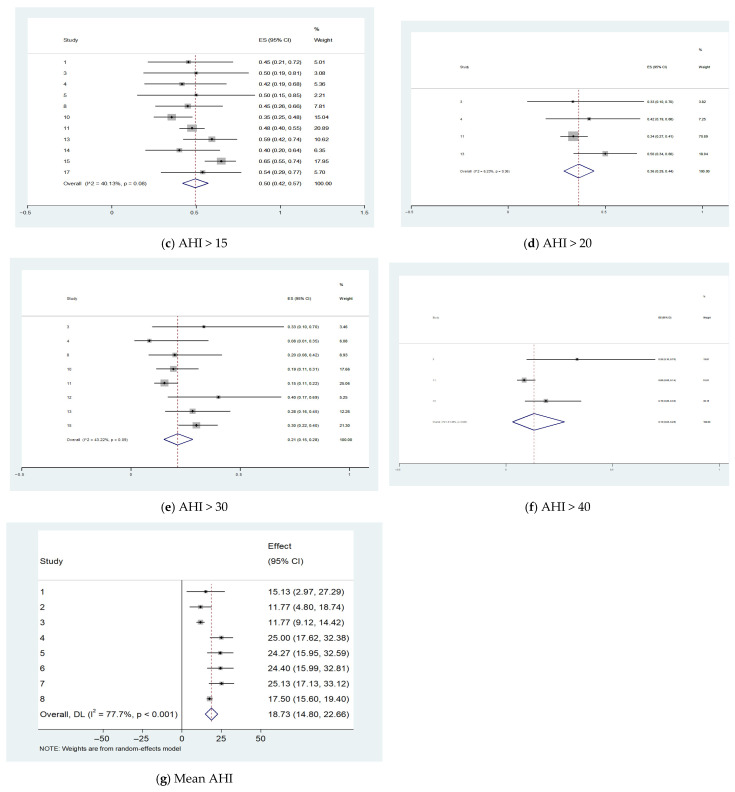
Forest plot of prevalence of SDB in ICH and mean AHI. Forest plots displaying the pooled prevalence of SDB among ICH survivors across escalating apnea–hypopnea index (AHI) thresholds and mean AHI: (**a**) Prevalence of SDB with AHI > 5 events/h; (**b**) prevalence of SDB with AHI > 10 events/h; (**c**) prevalence of SDB with AHI > 15 events/h; (**d**) prevalence of SDB with AHI > 20 events/h; (**e**) prevalence of SDB with AHI > 30 events/h; (**f**) prevalence of SDB with AHI > 40 events/h; (**g**) mean AHI (events/h) [1,2,3,4,5,6,7,8,9,10,11,12,13,14,15,16,17].

For AHI > 10, 12 studies comprising 346 patients showed that 64% (95% CI: 56% to 72%) met this threshold, with similar moderate heterogeneity (*I*^2^ = 47.4%, *p* = 0.03). For AHI > 15, based on 11 studies with 431 patients, the prevalence was 49% (95% CI: 42% to 57%), with moderate heterogeneity (*I*^2^ = 44.8%, *p* = 0.05). The prevalence for AHI > 20 was 37% (95% CI: 30% to 44%) across four studies, with low heterogeneity (*I*^2^ = 4.1%, *p* = 0.37), suggesting more consistent estimates among studies at this threshold. For SDB (AHI > 30 events/h), pooled data from eight studies showed a prevalence of 21% (95% CI: 15% to 27%), with moderate heterogeneity (*I*^2^ = 44.9%, *p* = 0.08). At AHI > 40, only three studies were available, yielding a prevalence of 13% (95% CI: 3% to 23%) with moderate heterogeneity (*I*^2^ = 43.1%, *p* = 0.17). An exploratory mean analysis across available studies reported a weighted average AHI of 19.08 events/h (95% CI: 14.78 to 23.39), though considerable heterogeneity was observed (*I*^2^ = 86%, *p* < 0.01) (Figure 2, Table 4).

Collectively, these findings demonstrate a high burden of SDB among ICH survivors, with over half of patients exceeding clinically relevant AHI thresholds, and a notable proportion exhibiting moderate-to-severe SDB (AHI > 15 or > 30 events/h). Despite some variability across studies, heterogeneity tended to decrease at higher severity thresholds, suggesting more consistent identification of severe SDB cases.

### 4.2. OSA and CSA

From pooled analysis of 10 studies, the prevalence of obstructive sleep apnea (OSA) among intracerebral hemorrhage (ICH) survivors was 73% (95% CI: 64% to 82%), with moderate heterogeneity observed (*I*^2^ = 41.3%, *p* = 0.08) (Table 4).

The pooled prevalence of central sleep apnea (CSA) across three studies was 5% (95% CI: 2% to 9%), with low heterogeneity (*I*^2^ = 7.2%, *p* = 0.34), indicating minimal variability between studies. The prevalence of OSA among the subset of studies that also reported CSA data was 70% (95% CI: 54% to 87%), based on four studies including 209 patients, with moderate heterogeneity (*I*^2^ = 64.2%, *p* = 0.04).

Using the second timing framework (<7 days, 7–30 days, >30 days), CSA prevalence within 7 days was 4% (95% CI: −2% to 10%), with no change beyond 30 days (6%, 95% CI: 3% to 11%, *I*^2^ = 0%, *p* = 0.56). OSA prevalence showed a stepwise temporal evolution, with 59% (43% to 74%) within 7 days, 75% (30% to 95%) between 7 and 30 days, and 82% (76% to 87%) beyond 30 days (*I*^2^ = 64.2%, *p* = 0.02 for subgroup differences). These findings indicate that while CSA prevalence remained low and stable over time, OSA was common post-ICH, with prevalence increasing beyond the acute phase

Pooled estimates were calculated using both risk ratio (RR) and odds ratio (OR) models to quantify the relative likelihood of OSA compared to CSA. The pooled risk ratio (RR) for OSA versus CSA was 7.44 (95% CI: 2.79 to 19.81), indicating that patients with OSA were over seven times more likely to be observed among ICH survivors with SDB compared to those with CSA. Moderate heterogeneity was present (*I*^2^ = 56.1%, *p* = 0.077).

**Figure 3:**
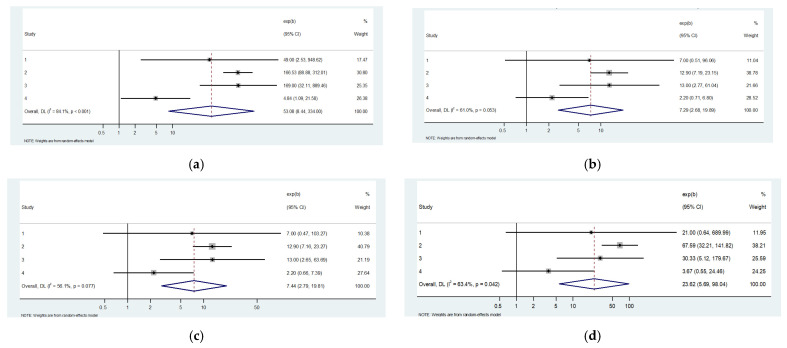

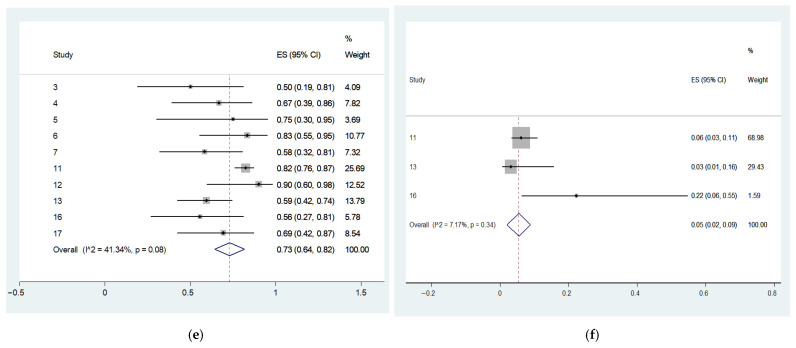
Prevalencem relative risk and odds ratios of OSA and CSA. (**a**) OR in ICH survivors with SDB (top left); (**b**) RR in ICH survivors with SDB (top right); (**c**) OR in ICH survivors (middle left); (**d**) RR in ICH survivors (middle right); (**e**) prevalence of OSA in ICH survivors (bottom left); (**f**) prevalence of CSA in ICH survivors (bottom right) [1,2,3,4,5,6,7,11,12,13,16,17].

Similarly, the pooled odds ratio (OR) for OSA versus CSA was markedly elevated at 53.08 (95% CI: 8.44 to 334.00), reflecting substantially higher odds of OSA relative to CSA within this population. However, considerable heterogeneity was observed (*I*^2^ = 84.1%, *p* < 0.001) (Figure 3).

The results consistently demonstrate a strong association between OSA and ICH compared to CSA, with both RR and OR estimates suggesting significantly greater prevalence of OSA among affected patients. While heterogeneity was moderate to high, particularly in the OR analysis, the overall direction and magnitude of the association remain robust.

### 4.3. Subgroup Analysis

At AHI > 5, prevalence peaked when testing was conducted 30 days to 3 months post-ICH (88%; 95% CI: 83–93%), compared with earlier assessments (<30 days: 85%; 78–93%) or later evaluations (>3 months: 67%; 39–86%), with moderate heterogeneity (*I*^2^ = 47.5%; heterogeneity *p* = 0.05; between-group *p* = 0.29). Further stratification demonstrated persistently high prevalence both in the acute window (<7 days: 82%; 67–96%) and subacute period (7–30 days: 86%; 78–93%), with no significant between-group differences across early timing strata (between-group *p* = 0.73), supporting a sustained burden of SDB in the early phases following ICH. Prevalence differed by patient location, with higher rates in inpatient and rehabilitation settings compared with home-based assessments (between-group *p* = 0.26) and was significantly elevated in studies from Asia and North America relative to Australia or Europe (continent-level between-group *p* = 0.07).

At AHI > 10, prevalence again remained highest between 30 days and 3 months (69%; 60–79%), with lower rates observed <30 days (62%; 48–76%) and beyond 3 months (58%; 39–78%). Although heterogeneity was moderate overall (*I*^2^ = 47.4%; *p* = 0.03), between-group differences by timing were not statistically significant at this threshold (between-group *p* = 0.51). However, when early assessments were further stratified, prevalence was significantly higher in the acute phase (<7 days: 71%; 48–93%) compared with the subacute 7–30-day window (51%; 41–61%), with significant between-group heterogeneity (between-group *p* = 0.02). Differences by hypopnea criteria were also significant (between-group *p* = 0.02), while subgroup comparisons by device type, continent, and study design were non-significant (between-group *p* > 0.05).

For AHI > 15, SDB prevalence demonstrated a similar temporal pattern, highest within the first 7 days (63%; 55–71%), lower between 7 and 30 days (38%; 29–48%), and intermediate beyond 30 days (47%; 41–54%). Overall heterogeneity was moderate (*I*^2^ = 44.8%; *p* = 0.05), though between-group differences by timing were not statistically significant (between-group *p* = 0.86 for Timing 1; *p* = 0.94 for Timing 2). Regional variability persisted, but continent-level differences did not reach statistical significance (between-group *p* = 0.17). Differences by device type, hypopnea criteria, and study design similarly showed non-significant between-group comparisons (all *p* > 0.05).

With increasing severity thresholds, overall SDB prevalence declined, but early timing remained associated with higher prevalence. At AHI > 20, prevalence was higher within 30 days (47%; 31–63%) compared with beyond 30 days (34%; 27–41%), particularly within the first 7 days, although between-group differences by timing were not statistically significant (between-group *p* = 0.29 and *p* = 0.14, respectively). Overall heterogeneity was low (*I*^2^ = 6.2%; *p* = 0.36), and subgroup comparisons across diagnostic approach, continent, and study design were non-significant (all *p* > 0.05).

Similar findings were observed at AHI > 30, where early assessments (<7 days: 30%; 23–38%) yielded higher prevalence compared with later time points (7–30 days: 20%; 11–28%; >30 days: 14%; 9–20%), with significant between-group differences by timing (between-group *p* = 0.01). Overall heterogeneity remained moderate (*I*^2^ = 43.2%; *p* = 0.09), and differences by study design were also significant at this threshold (between-group *p* = 0.01), while device type and hypopnea criteria were not.

At the most severe threshold (AHI > 40), overall prevalence was lowest (13%; 3–28%) but remained higher for assessments within 7 days (20%; 8–33%) compared with beyond 30 days (9%; 5–14%), with significant between-group differences by timing and study design (between-group *p* = 0.03). Heterogeneity was moderate (*I*^2^ = 61.4%; *p* = 0.08), and subgroup differences by hypopnea type and continent approached but did not consistently reach statistical significance.

**Figure 4:**
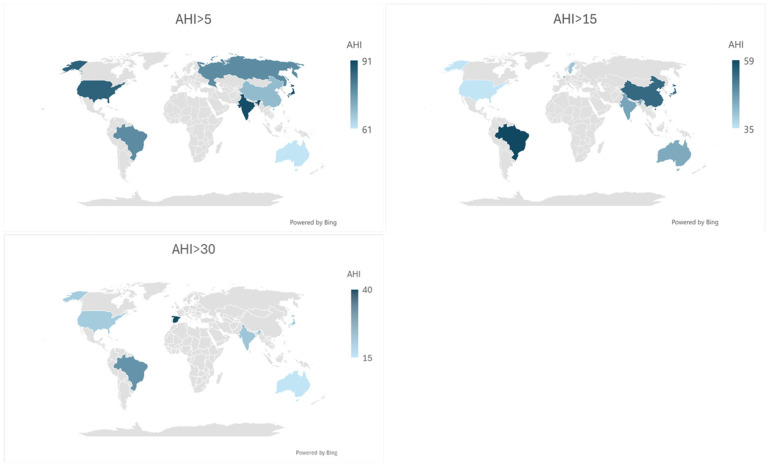
Maps of prevalence of sleep-disordered breathing severity by country.

Overall, across AHI thresholds, SDB prevalence was significantly influenced by timing of assessment, patient location, continent, diagnostic approach, and study design, with the early and subacute post-ICH periods representing windows of heightened vulnerability, particularly at higher severity thresholds (Figure 4; Supplementary Materials S3).

### 4.4. Prognostic Factors of SDB Associated with ICH

From the meta-analysis, there were five studies [14,26,29,42] from which age BMI and presence of hypertension could be extracted: The mean age was 62.23 years (95% CI: 56.72–67.7); and the mean BMI was 27.64 (95% CI: 23.87–31.41). SDB is linked with increased ICH severity [29]. Men were more likely to have SDB after ICH.

Consistent with the existing literature, hypertension was a common pathology among ICH patients who had SDB in this meta-analysis [2]. In four studies reporting hypertension prevalence among ICH patients, the pooled prevalence was 96% (95% CI: 85–100%; *I*^2^ = 73%, *p* = 0.01). Meta-regression showed no association between hypertension prevalence and SDB rates (coefficient = 0.005, 95% CI: −0.023 to 0.034; *p* = 0.72).

Additional prognostic variables, including ICH location and temporal changes in SDB severity, were evaluated qualitatively in a subset of studies. Furthermore, SDB has been shown to improve over time [52], with some literature showing a significant reduction in central apneas but not obstructive apneas after stroke [20]. Limited data on demographic variables such as ethnicity were available in a few studies [7]. Many of the studies did not have separate parameters on ICH patients and ischemic stroke patients and stated them as pooled data; these studies had a significantly larger number of patients with ischemic stroke, and thus, estimation or conclusions about these parameters could not be established.

## 5. Discussion

This meta-analysis demonstrates that sleep-disordered breathing (SDB) is highly prevalent among survivors of intracerebral hemorrhage (ICH), with more than 85% of patients meeting diagnostic criteria at an apnea–hypopnea index (AHI) threshold of >5 events per hour and approximately half exhibiting moderate-to-severe disease. Substantial heterogeneity in prevalence was observed across studies, influenced by timing of assessment, clinical setting, diagnostic modality, and geographic region, with higher rates reported in early post-ICH evaluations and inpatient or rehabilitation settings. Obstructive sleep apnea emerged as the predominant SDB phenotype, supporting its role as a potential risk factor for ICH, whereas central sleep apnea appears more likely to arise as a consequence of brain injury. Despite the near-universal presence of hypertension among ICH patients, meta-regression did not identify a significant association between hypertension prevalence and SDB burden, suggesting that additional factors may contribute. Collectively, these findings highlight SDB as a common and clinically meaningful complication following ICH, with plausible pathophysiological mechanisms that may influence neurological injury and recovery.

### 5.1. Development of SDB in Patients with ICH

The bidirectional relationship between SDB and ICH is apparent; however, it is unclear whether SDB more commonly precedes ICH or occurs as a result of it. One study demonstrated that up to 33% of ICH patients had a high likelihood of SDB through screening questionnaires [6,7]. The pathophysiology and mechanism of injury are multifactorial in ICH and SDB, and several variables have been reported both as risk factors and outcomes of ICH. Shibazaki found patients with ICH were significantly more likely to develop dysphagia and dysphasia [29]. Pontes et al. found that snoring was more common in ICH survivors with AHI > 10 (60% vs. 16.7%; *p* = 0.02). Dysfunction of the pharyngeal muscles can lead to impairment of swallowing as well as upper airway resistance and thus SDB [29]. One study demonstrated the odds of developing SDB was 4.5 times higher for hemorrhagic stroke compared to ischemic stroke (OR 4.5 [1.2–16.8]) [41]. Other studies have found mixed results (Table 3) [10,20].

### 5.2. Primary Brain Injury

Initial hemorrhage, continued hemorrhage, and hematoma expansion result in mass effect and mechanical disruption from extravasated blood. This can lead to primary brain injury due to increased global pressure (increased intracranial pressure). Ischemia may occur due to herniation syndromes causing arterial compression due to a global increase in intracranial pressure (ICP). Mechanical compression of local structures may result from hematomas and may prove lethal when hemorrhage occurs in the posterior fossa. This can lead to local compression of the brain, which may lead to cardiorespiratory dysfunction and may play a role in the development of SDB. Greater neurological injury severity, as reflected by lower Glasgow Coma Scale scores, has been associated with a higher prevalence of sleep-disordered breathing in patients with intracerebral hemorrhage.

ICH is attributed to spikes in blood pressure and therefore is considered to be rare during the asleep period when blood pressure is normally lower and is also associated with decreased hematoma expansion [53]. Anticoagulation drugs have been implicated in the development of ICH. ICH cases associated with vitamin K antagonists (such as coumadin) have increased hematoma volume, rates of expansion, and ventricular extension, all of which are associated with poor prognosis [54,55,56]. Male gender has also been found to be associated with increased hematoma expansion, most likely attributable to higher prevalence of hypertension [8]. Involvement of intraventricular hemorrhage is associated with a higher risk of mortality and is more prevalent in men, while lobar hemorrhage is more prominent in women [3,8]. OSA increases blood pressure, which is associated with worse outcomes, highlighting the importance of prevention, diagnosis, and early treatment of SDB in decreasing incidence or recurrence of ICH or other cardiovascular events.

### 5.3. Secondary Brain Injury

Secondary brain injury is primarily due to perihematomal edema (PHE), which is edema around the primary brain injury (hematoma) [26,57]. It can occur within hours of ICH event and may last for days or weeks [11]. It is thought to evolve most rapidly over the first 1–3 days and starts to regress after a period of 10–14 days; however, it can last up to 3 weeks [26]. Increased PHE and SDB are both associated with cognitive and functional impairment [48,58]. PHE is known to occur in over 33% of patients on the first day post-ictus. In one study, patients with severe apnea (defined as AHI ≥ 30) had more PHE (RPHE) (56.24 ± 26.6 vs. 37.8 ± 19.60; *p* = 0.029) relative to those with minor or no SDB [26]. PHE is directly related to severity of SDB. One study found that after adjusting for multiple variables, there was a positive correlation between AHI and RPHE volume that was lower at baseline [Pearson correlation coefficient (rs): 0.40; *p* = 0.030], compared to 24 h (rs = 0.46; *p* = 0.011), and increased to moderate on days 4–5 after the ICH event (rs = 0.59; *p* = 0.006) [26]. The total white blood cell count compared at admission compared to on days 4–5 had a positive correlation with relative edema volume (rs = 0.57; *p* = 0.008). Studies have shown that neutrophil count is inversely related to edema expansion, while monocytes and eosinophils are associated with an increase, especially the latter [59].

Secondary brain injury (PHE) is a result of the physiological response to hematoma and biochemical and metabolic byproducts of the clotting cycle [57]. Thrombin, erythrocyte components, and their degradation products, increased hydrostatic pressure, and disruption of the blood–brain barrier result in brain edema [2,60]. Inflammation and blood product toxicity (hemoglobin and iron) also contribute to injury [60]. Recent translational research has identified possible therapeutic targets in inflammation and signaling pathways involved with microglial activation, red blood cell lysis, white blood cell infiltration, and toll-like receptor activation (TLR) [61,62,63,64]. It is thought that the penumbra (region surrounding the area of insult) may have impaired mitochondrial oxygenation, resulting in a metabolic crisis with reduction in oxygen use, thus impairing function, perhaps contributing to SDB as well. Neuronal death and brain atrophy can result from mechanic forces during hematoma formation or blood clots and degradation products [65]. Resolution of hematoma, edema reduction, neuroplasticity, and neurogenesis may lead to improved functional recovery and thus improvement of SDB [2,66]. The variability in SDB severity across time periods after ICH in subgroup analysis may be influenced by mechanisms of primary or secondary brain injury. Further evaluation of methods targeting the aforementioned factors through both translation and clinical research are necessary to evaluate for potential contributions to the development and treatment of ICH and SDB.

### 5.4. SDB Role in ICH

OSA is associated with intermittent hypoxemia and recurrent sympathetic arousals which lead to an increased multitude of comorbidities including hypertension coronary artery disease, heart failure, arrythmias, pulmonary hypertension, and stroke [67,68]. Decreased ventilation results in oxygen desaturation (chronic intermittent hypoxemia), leading to sympathetic nervous system activation and sustained hypertension, both nocturnally and during the day [69]. It also leads to oxidative stress because of cerebral hemodynamic dysfunction due to endothelial dysfunction and inflammation. These contribute to atherogenic and prothrombotic states and dysregulation of the cerebral circulation. This further predisposes to suboptimal repair capacity of the vascular endothelium, adverse cardiovascular outcomes and impaired recovery of function. Fewer studies examine CSA roles in ICH; however, it is thought that CSA may be a sign of silent cerebral infarction manifesting as dysregulation of central respiratory mechanisms [7,70]. This may result in hypertension and thus, increasing the risk of ICH as well; further exploration is needed in the complexities of the relationship between CSA and ICH.

### 5.5. Transience

Transience of sleep-disordered breathing (SDB) in patients with intracerebral hemorrhage (ICH) has been reported [19]. The literature has demonstrated that patients experienced significantly less frequency and severity of SBD after the acute phase compared to during the acute phase [19], especially in posterior circulation ICH [19]. Other studies have reported contrasting findings, which may be attributable to the presence of pre-existing SDB prior to stroke onset [71]; improvement in SDB has been observed after three months, characterized by a significant reduction in central apneas but persistence of obstructive events [19]. In addition, a subgroup meta-analysis involving relatively homogeneous populations and methodologies demonstrated a decrease in both SDB prevalence and severity from the acute to subacute stroke period [14].

In subgroup analyses, a decrease in SDB severity over time was observed. Changes in cerebral blood flow velocity have been shown to precede apneic events and associated blood pressure fluctuations in CSA [23]. In hemorrhagic stroke, SDB appears more likely to be central in origin and transient compared with ischemic stroke, suggesting that OSA may more commonly precede ICH, whereas CSA may arise as a consequence of acute brain injury [19,72]. The severity of SDB may reflect acute neurological dysfunction, as increases in SDB severity have been associated with greater perihematomal edema (PHE) [26]. Although transience has also been reported in ischemic stroke-related OSA, studies consistently demonstrate that CSA resolves more rapidly and is more frequently transient in ICH populations [19,72]. SDB may be attributable to the acute dysfunction and regression of hematoma and edema, as increases in severity of SDB are thought to occur with increases in PHE [26]. Assessment of temporality remains challenging, as many patients are ineligible for early evaluation and experience high early mortality or loss to follow-up.

### 5.6. Oropharyngeal Muscle Dysfunction

Oropharyngeal muscle dysfunction causes SDB. The largest pharyngeal muscle, the genioglossus, is innervated by the hypoglossal nerve, which originates from the brainstem [73] and acts to pull the tongue forward. SDB patients experience a reduction in genioglossus activation during sleep, which importantly contributes to upper-airway collapsibility. Similarly, other lingual muscle dysfunction, such as styloglossus hyoglossus muscle dysfunction, and the effects of gravity play a role in obstruction and decreased airway patency as well. For this reason position during sleep can cause or worsen SDB [14]. Studies have shown that brainstem strokes affect pharyngeal muscle activity [29] resulting in dysphagia and reduced upper-airway patency, thereby increasing vulnerability to sleep-disordered breathing. Additionally oropharyngeal morphology, including lingual anatomy, may also be associated with SDB [47,74].

Dysfunction in other oropharyngeal muscles such as the soft palate muscles, pharyngeal constrictors, suprahyoid muscles, and laryngeal muscles have been linked to SDB [75]. As oropharyngeal muscle weakness can be transient, so can sleep apnea after ICH. Studies have shown that starting rehabilitation can decrease AHI and lead to faster recovery [76,77]. Transience can be difficult to assess due to patients being ineligible for evaluation at initial presentation and a high mortality or loss to follow.

A study found a higher association of dysphagia and dysarthria in severe SDB compared to mild SDB [dysphagia (76% vs. 51%, *p*-value 0.006)]; dysarthria (76% vs. 42%, *p*-value 0.025); dysarthria and dysphagia together (76% vs. 32%, *p*-value 0.008), which was attributed to prevalence of facial features and upper way anatomy. Phenotypic anatomical variation, such as dolichofacial morphology, has been associated with increased susceptibility to SDB [78]. Autonomic factors, including altered baroreflex sensitivity, may further modulate disease severity and downstream cardiovascular consequences, rather than primary SDB development [23]. Advancements in genomics and translational medicine involving the pathways of pathogenesis in ICH and SDB may prove beneficial.

### 5.7. Cerebral Injury Location

Cerebra injury location may contribute to higher prevalence and severity of SDB observed in stroke patients. The cerebral injury location was not found to be statistically significant in severity or development in many studies [51,72]. One univariate analysis showed that individuals with OSA had higher relative prevalence of lobar hemorrhage (27% vs. 10%, *p*-value 0.038). After a multivariate regression, lobar hemorrhage was associated with increased odds of OSA (OR = 5.339 [95% CI: 1.399–20.376]; *p* = 0.014) and negatively with diabetes mellitus (OR = 0.235 [95% CI: 0.063–0.871]; *p*-value 0.03) when adjusting for BMI, hypertension, and age [71]. The highest lobar involvement for OSA was as follows: frontal, parietal, temporal, and occipital lobes [14]. This study suggested that airway obstruction in OSA patients affects the frontal lobe, which controls the muscles of the pharynx, the dysfunction of which leads to upper airway obstruction. Increased sympathetic activity along with lower relative tolerance to hypoxic exposure and dysregulation of vascular endothelial function results in cerebral bleeding [71]. Although theoretically increased size of ICH is correlated with increased risk of SDB, there is a large variation in location and size, with a limited number of patients to study, providing inconclusive results. Further pooled data is needed to establish a relationship between the location and size of ICH lesions with SDB.

### 5.8. Positional Apnea

Positional sleep apnea is defined as when individuals’ AHI is at least two times greater in a lateral position compared to their supine AHI [14]. Sleeping in a supine position is believed to increase the amount and severity of apneic events [14]. One study highlighted the increase in SDB in the supine position compared to the lateral position and attributed the increased collapsibility of the upper airways to it. This study showed that 21% of all stroke patients developed positional OSA (POSA) [14]. The same study showed 25% of ICH patients developed POSA and 71% possibly developed it [14]. Out of the five ICH patients with confirmed OSA, the mean AHI while supine was 32.7 ± 22 and the mean AHI while lateral was 4.9 ± 2.5 (*p*-value < 0.001) [14]. This study also showed that 75% of patients with ICH who were tested for SDB, spent all sleep time in a supine position [14]. Many patients, especially those in critical condition, spend the majority of their time sleeping in a supine position, which increases the difficulty of assessment, and about which there is limited published data from existing studies. A lateral sleeping position may prevent the development of episodes of apnea or hypopnea. When various demographic factors and possible cognitive impairment in patient populations are taken into consideration [14], it may be difficult to enforce positional guidelines adequately in both the acute and post-acute stroke period. Exploration and implementation of strategic positioning in acute ICH patients, especially those in critical states (as they are more prone to supine positioning), are needed and may prove beneficial.

### 5.9. Ethnicity and Socioeconomic Factors

The literature has reported a higher prevalence of SDB and ICH amongst certain racial and ethnic groups including African Americans, Latinos/Hispanics, Native Americans, and East Asians [7,29,48,78,79]. This has been attributed to socioeconomic and genetic factors as well as the prevalence of hypertension, diabetes, and microvascular cerebral disease. A study reported a higher prevalence of SDB in ICH in Mexican Americans (MA) compared to Caucasian Americans, potentially due to sociodemographic and genetic factors [7]. MA had a higher comparative prevalence of excessive alcohol intake (16.6% vs. 7.1%, *p*-value: 0.03); initial GCS of 14.0 (95% CI: 7.0, 15.0) vs. 24.7 (95% CI: 21.6, 29.3; *p*-value 0.005); and BMI: 15.0 (95% CI: 11.0, 15.0) vs. 28.3 (95% CI: 25.5, 32.9), *p*-value < 0.001 [7]. As these risk factors were higher in ICH survivors who did not participate in screening, the prevalence was likely higher.

### 5.10. Future Directions

Despite the high burden of sleep-disordered breathing (SDB) following intracerebral hemorrhage (ICH), systematic screening remains uncommon. The critical condition of many ICH patients frequently precludes in-laboratory polysomnography and, in some cases, even conventional portable sleep apnea testing, underscoring the need for pragmatic, minimally intrusive screening strategies. Emerging evidence supports the utility of portable devices and oximetry-based approaches, particularly for detection of moderate-to-severe SDB [80,81,82].

Recent advances in artificial intelligence-enabled, non-invasive wearable technologies capable of continuous cardiorespiratory and oxygen saturation monitoring offer a promising avenue to address these limitations [83,84]. Such technologies may allow SDB screening to be integrated across the full continuum of ICH care, including acute settings (ICU and inpatient wards), post-acute environments (rehabilitation facilities and nursing homes), outpatient clinic settings, and post-discharge home monitoring [81,82]. By enabling earlier identification of clinically significant SDB—often during periods when traditional testing is not feasible—these approaches may facilitate timely initiation of targeted interventions, longitudinal monitoring of disease transience or persistence, and optimization of secondary prevention strategies.

Implementing minimally intrusive, AI-assisted screening across care transitions has the potential to improve recognition and management of SDB in ICH survivors, reduce missed diagnoses in the most vulnerable phases of recovery, and ultimately support better neurological and cardiovascular outcomes. Future prospective studies are needed to validate these technologies in ICH populations, define optimal screening thresholds, and determine their impact on functional recovery and long-term outcomes.

## 6. Limitations

Several limitations should be acknowledged. Factors related to the acute severity of intracerebral hemorrhage hinder the ability to study sleep-disordered breathing in this population. As a result, the true prevalence of sleep-disordered breathing is likely underestimated due to selection bias arising from outcome measurement [26]. This concern is supported by studies reporting that non-participants had worse overall health status, suggesting systematic exclusion of more severely ill patients. Consequently, critically ill or highly acute patients are underrepresented in sleep-disordered breathing assessments, likely leading to underestimation of both prevalence and severity in the highest-risk groups.

Assessment of SDB at varying time points following ICH further complicates interpretation, as evolving neurologic and physiologic changes across acute, subacute, and chronic phases may confound observed associations. In addition, heterogeneity in diagnostic devices and scoring criteria may contribute to misclassification bias, particularly in milder forms of SDB, where underdiagnosis is more likely.

Incomplete reporting of key clinical variables and comorbidities associated with both ICH and SDB, combined with generally small sample sizes, limited the ability to perform comprehensive subgroup analyses. Missing data and lack of stratification prevented evaluation of several potentially relevant associations. Small study sizes also reduce statistical power and may contribute to imprecision in prevalence estimates and effect measures, particularly for comparisons between obstructive and central sleep apnea. Smaller studies may additionally be prone to overestimation of effects; therefore, they must be interpreted cautiously. Risk of bias varied across studies, with most showing low to moderate concerns. Common limitations included unclear reporting of participant selection, exposure classification, and missing data, reflecting challenges in SDB research among ICH survivors.

Methodologically, study screening and data extraction were conducted by a single reviewer, which may increase the risk of selection or classification bias, although standardized eligibility criteria and predefined extraction fields were applied to mitigate this risk. In addition, the review protocol was not prospectively registered, which may limit transparency but does not affect the conduct or reporting of the analyses.

Risk of bias varied across studies but was generally low to moderate. Common methodological limitations included unclear participant selection processes, exposure classification, and incomplete reporting of covariates, reflecting broader challenges in conducting sleep research among ICH survivors.

Assessment of publication bias revealed borderline small-study effects at AHI > 5 (Egger’s *p* = 0.08) and significant bias at AHI > 40 (Egger’s *p* = 0.027), suggesting selective reporting at lower and extreme severity thresholds. No evidence of small-study effects was detected for AHI thresholds between >10 and >30. For the obstructive versus central sleep apnea analysis, no publication bias was identified (Egger’s *p* > 0.74), supporting the relative robustness of this comparison.

Overall, despite these limitations, the principal findings—namely the high burden of SDB and the predominance of obstructive sleep apnea following ICH—were consistent across analyses. Nevertheless, results should be interpreted cautiously, and future studies with standardized assessment protocols, larger sample sizes, and more complete covariate reporting are needed to refine prevalence estimates and clarify mechanistic associations.

## 7. Conclusions

SDB is highly prevalent among ICH survivors, with over 85% meeting diagnostic criteria at AHI > 5 and approximately half experiencing moderate-to-severe SDB. OSA is the predominant phenotype, though CSA may occur, particularly as a consequence of injury. Subgroup variability highlights the influences of timing, geography, and diagnostic methods on reported prevalence.

These findings emphasize the critical need for routine SDB screening in ICH survivors, especially those with risk factors such as obesity, male sex, or early neurological impairment. Development of pragmatic screening protocols utilizing portable or limited-channel devices, alongside targeted treatment of modifiable contributors like hypertension, may help mitigate long-term neurological and cardiovascular morbidity in this high-risk population. Further research is necessary to delineate the temporal evolution of SDB post-ICH, assess the impact of treatment, and explore underlying mechanisms linking SDB to ICH severity and recovery.

## Figures and Tables

**Table 1:** Characteristics of studies.

Study	Published	Study Type	Country	Location	Time of Assessment	Positive Criteria	Patients with SDB	ICH Survivors	Timing	Scored	Diagnosis	Pre-Screening Questions	Hypopnea AASM	Hypopnea Criteria	Device Type AASM/	Age	BMI	Hypertension
**Aaronson [38]**	2012	Retrospective	Netherlands	Rehab Unit	May 2007 to July 2012	AHI ≥ 15	5	11	Within 2 M average time 30 D ± 27.3	Manually	CT or MRI	ESS	2	B	3			
**Boulos [39]**	2017	Prospective	Canada	Inpatient (S) and Outpatient	July 2014–June 2015	AHI ≥ 10	4	7	33.5 D	Manually			2	A	4			
**Broadley [40]**	2007	Prospective	Australia	Inpatient (S)	2001 (4-month period)	AHI ≥ 10	3	6	3–4 D	Manually	CT and MRI	ESS	7	B	3			
**Cadilhac [41]**	2005	Prospective	Australia	Home	Oct 1998 to Sept 1999	AHI ≥ 5	8	12	3 Y	Manually	CT	ESS	3	-	3			
**Dyken [42]**	1996	Prospective	USA	Inpatient (S)	Before 1996	AHI ≥ 5	3	4	26 D	-	CT	-	2	A	1	64.5	33.18	75
**Kaneko [43]**	2003	Prospective	Canada	Rehab Unit	-	AHI ≥ 10	10	12	44.8 D ± 3.1	Automatic	CT or MRI	ESS	4	-	1			
**Katzan [44]**	2016	Retrospective	USA	Sleep Lab	Jan 2011 to Dec 2012	AHI > 10	7	12	1 Y	Manually	-	STOP	1	A	1			
**Kumar [13]**	2017	Prospective	India	Sleep Lab	-	AHI ≥ 5	18	20	>1 W	Manually	Neuro-imaging	ESS	1	A; B	1			
**Lefèvre-Dognin [45]**	2014	Prospective	France	Rehab Unit	-	AHI ≥ 10	5	10	26 D	Manually	-	ESS			4			
**Lisabeth [7]**	2018	Prospective	USA	Cohort	July 2010 to May 2016	REI ≥ 10	29	62	11 D	Manually	-	BSQ; Other	2	B	3			
**Matsuura [46]**	2019	Retrospective	Japan	Rehab Unit	August 2011 to November 2013	REI ≥ 15	78	164	45 D (13.1)	-	CT or MRI	ESS	2	A	3	64	29.27	85.3
**Parra [47]**	2000	Prospective	Spain	Inpatient (S)	1986 to 1991	AHI > 10	9; 3	10; 4	2–3 D/ 2nd 2 M	Manually	CT and MRI	ESS; Other	3	A	3	73	24.8	
**Pontes [26]**	2009	Prospective	Brazil	Inpatient	Jan 2006 to Jan 2008	AHI ≥ 10	20	32	1–2 D	Manually	CT	Other	5	A	1	57	26.5	100
**Sandberg [12]**	2001	Prospective	Sweden	Rehab Unit	April 1995 to May 1997	AHI ≥ 10	9	15	23 D ± 8	Manually	CT or Mental Status	-	1	A	3			
**Shibazaki [29]**	2013	Prospective	Japan	Inpatient	2010 to 2013	RDI ≥ 5	91	97	5 D (1–7)	Manually	CT or MRI	JESS [48]; Other	2	B	3	68.71	23	96
**Tazartukova [49]**	2018	Prospective	Russia	Inpatient (I)	-	AHI ≥ 5	7	9	4 D	Manually	CT or MRI	-	1	A	2			
**Zhang [48]**	2017	Prospective	China	Sleep Lab	June 2013 to June 2015	AHI ≥ 5	9	13	6 W	-	-	ESS	2	A	1			

Sleep study type: F. Full polysomnography (including EEG); L, Limited (without EEG); Time D: day, W: Week, Y: Year. ESS: Epworth Sleepiness Scale; JESS: Japanese Epworth Sleepiness Scale; BSQ: Berlin Sleep Questionnaire; SSS: Stanford Sleepiness Scale; STOP: STOP questionnaire. Location: Inpatient Unit, Inpatient S (Stroke Unit); Inpatient I (Intensive Care Unit); Home: Home-based study; Sleep Lab: Sleep laboratory; Rehab Unit: Stroke Rehabilitation Unit; Cohort: Hospital, rehabilitation setting, nursing home, or home; Timing: Time from stroke to SDB testing. Hypopnea AASM: 1: Reduced flow with arousal or desaturation. 2: Reduced flow with desaturation. 3: Reduced flow or reduced effort with desaturation. 4: Reduced flow. 5: Reduced effort with desaturation or arousal. 6: Reduced flow or effort with desaturation or arousal. 7: Other. Hypopnea criteria: (A) Desaturation ≥ 3% from baseline. (B) Desaturation ≥ 4% from baseline.

**Table 2:** Sleep-disordered breathing in intracerebral hemorrhage survivors by severity *n* (%).

Study	AHI > 5	AHI > 10	AHI > 15	AHI > 20	AHI > 30	AHI > 40
**Aaronson [38]**	-	-	5 (45)	-	-	-
**Boulos [39]**	-	4 (57)	-	-	-	-
**Broadley [40]**	3 (50)	3 (50)	3 (50)	2 (33)	2 (33)	2 (33)
**Cadilhac [41]**	8 (67)	7 (58)	5 (42)	5 (42)	1 (8.3)	-
**Dyken [42]**	3 (75)	3 (75)	2 (50)	-	-	-
**Kaneko [43]**	-	10 (83)	-	-	-	-
**Katzan [44]**	-	7 (58)	-	-	-	-
**Kumar *^a^* [13]**	18 (90)/17 (85)	-	9 (45)/9 (45)	-	4 (20)/3 (15)	-
**Lefèvre-Dognin [45]**	-	5 (50)	-	-	-	-
**Parra *^b^* [47]**	-	9 (90)/3 (75)	-	-	4 (40); 3(75)	-
**Pontes [26]**	25 (78)	20 (63)	19 (59.4)	16 (50)	9 (28)	6 (18.75)
**Sandberg [12]**	-	9 (67)	6 (40)	-	-	-
**Tazartukova [49]**	7 (78)	-	-	-	-	-
**Zhang [48]**	9 (69)	-	7 (54)	-	-	-
	**REI > 5**	**REI > 10**	**REI > 15**	**REI > 20**	**REI > 30**	
**Lisabeth [7]**	52 (84)	29 (47)	22 (35)	-	12 (19)	
**Matsuura [46]**	145 (88.4)	110 (67.1)	78 (47.6)	55 (33.5)	25 (15.2)	14 (8.5)
	**RDI > 5**	**RDI > 10**	**RDI > 15**	**RDI > 20**	**RDI > 30**	
**Shibazaki [29]**	91 (94)	-	63 (65)	-	29 (26)	

AHI: Apnea hypopnea index; RDI: Respiratory disturbance index; REI: Respiratory event index. *^a^* Kumar et al., was assessed using desaturation threshold of ≥3% and ≥4%; *^b^* Parra et al., evaluated patients at two time points.

**Table 3:** Review of literature.

Study	Finding	Results	Clinical Significanc e
**Camilo ^a^ [14]**	Supine vs. lateral AHI	Supine AHI 32.7 ± 22 vs. lateral AHI 4.9 ± 2.5; *p* = 0.001	Supine position significantly worsens SDB
**Ishaq ^b^ [50]**	Transience of SDB and decreased severity and prevalence from acute to subacute stroke period	Mean AHI during acute stroke period: 24.4 (20.9 to 27.9) Mean AHI subacute during stroke period: 17.5 (15.6 to 19.49)	Temporal transience of SDB
**Lisabeth [7]**	WBC at admission correlated with edema (day 4–5)	*r* = 0.57; *p* = 0.008	Systemic inflammation linked to PHE volume
**Parra [47]**	Transience of CSA after ICH	CSA frequency/severity ↓ at 3 months; OSA unchanged	CSA may resolve post-ICH; OSA persists
**Pontes [26]**	AHI correlated with edema at baseline	*r* = 0.40; *p* = 0.030	SDB severity linked to acute edema burden
	AHI correlated with edema at 24 h	*r* = 0.46; *p* = 0.011	Relationship strengthens at 24 h
	AHI correlated with edema at day 4–5	*r* = 0.59; *p* = 0.006	Strongest correlation observed at day 4–5
	AHI > 30 associated with greater edema	56.2 ± 26.6 vs. 37.8 ± 19.6; *p* = 0.029	Severe SDB linked to increased edema
	Snoring more common in AHI > 10	60% vs. 16.7%; *p* = 0.02	Snoring predicts SDB severity
**Shibazaki [29]**	Waist circumference in RDI > 30 vs. <30	86 cm (84–92) vs. 84 cm (78–88); *p* = 0.019	Central adiposity linked to severe SDB
	Dysphagia/dysarthria in severe SDB	90% in RDI > 30 vs. 62% in RDI > 5; *p* = 0.008	Bulbar dysfunction common in severe SDB
	Diastolic BP in RDI > 30 vs. <5	104 (83–111) vs. 94 (80–107);	Possible diastolic BP elevation with severe SDB

↓: denotes decrease; ^a,b^: Not included in the meta-analysis as analyzed the same populations of other included studies.

**Table 4:** Percentage sleep disordered breathing in ICH Survivors.

Measure	# Studies (Patients)	% or Mean (95% CI)	Q	df	*I*^2^ (%)	*p*-Value
**AHI > 5**	10 (361/419)	0.85 (0.80 to 0.91)	17.14	9	47.48	0.05
**AHI > 10**	12 (217/346)	0.64 (0.56 to 0.72)	20.90	11	47.36	0.03
**AHI > 15**	11 (219/431)	0.49 (0.42 to 0.57)	18.10	10	44.76	0.05
**AHI > 20**	4 (78/214)	0.37 (0.30 to 0.44)	3.13	3	4.13	0.37
**AHI > 30**	8 (86/403)	0.21 (0.15 to 0.27)	12.71	7	44.92	0.08
**AHI > 40**	3 (22/202)	0.13 (0.03 to 0.23)	3.51	2	43.06	0.17
**Mean AHI**	8 (397)	19.08 (14.8–22.66)	31.43	7	77.7	<0.01
**CSA**	3 (10/205)	0.05 (0.03–0.09)	2.15	2	7.17	0.34
**OSA**	10 (361/419)	0.73 (0.64 to 0.82)	15.34	9	41.3%	0.08

Summary of pooled prevalence estimates for sleep-disordered breathing (SDB) at varying apnea–hypopnea index (AHI) severity thresholds (AHI > 5 to AHI > 40 events/h), mean AHI, and subtype-specific prevalence for central sleep apnea (CSA) and obstructive sleep apnea (OSA). All estimates reflect random-effects meta-analyses with corresponding 95% confidence intervals (CI), heterogeneity statistics (*I*^2^), and *p*-values.

## Data Availability

Data is not available for sharing.

## Supplementary Materials

The following supporting information can be downloaded at https://www.mdpi.com/article/10.3390/neurolint18010019/s1. Supplementary Table S1: PRISMA 2020 Checklist [85]; Supplementary Materials S1: PICO table; Supplementary Material S2: Search protocol [14,20,72,74,86,87,88,89,90,91,92,93,94,95,96,97,98,99,100,101,102,103,104,105,106,107,108,109,110,111,112,113,114,115,116,117,118,119,120,121,122,123,124,125,126,127,128]; Supplementary Materials S3: Percentage of Patients with SDB by subgroup; Supplementary Materials S4: Risk of Bias Summary.
