# Supplementary material for: Global Prevalence of Sleep-Disordered Breathing in Intracerebral Hemorrhage Survivors: A Meta-Analysis and Systematic Review
Source: Neurol Int. 2026 Jan 20;18(1):19. doi: 10.3390/neurolint18010019 (PMC12845411; doi:10.3390/neurolint18010019)
Supplement: Supplementary file 1 [file neurolint-18-00019-s001.zip › Supplementary Material S1. PICO table.pdf]

Table S2. PICO for Meta-Analysis of Sleep Disordered Breathing in Intracerebral Hemorrhage

**Population**	**Studies including intracerebral hemorrhage survivors (age 18 and above)**
**Intervention or exposure**	Studies that used American Association of Sleep Medicine approved devices (categories 1-4)
	Inclusion Criteria
	Observational or experimental studies
	Exclusion criteria
	Abstracts, case reports, meta-analysis, editorial, letter, protocol, conference poster, dissertation,
**Comparison**	Not applicable
**Outcome**	Prevalence of sleep disordered breathing-percentage of patient with AHI of >5, >10, >15, >20, >30, >40
	Mean AHI
