# Supplementary material for: Global Prevalence of Sleep-Disordered Breathing in Intracerebral Hemorrhage Survivors: A Meta-Analysis and Systematic Review
Source: Neurol Int. 2026 Jan 20;18(1):19. doi: 10.3390/neurolint18010019 (PMC12845411; doi:10.3390/neurolint18010019)
Supplement: Supplementary file 1 [file neurolint-18-00019-s001.zip › supplementary material S2. Search protocol-v3.pdf]

# I: Review Title

**Sleep Disordered Breathing in Intracerebral Hemorrhage: A Meta-Analysis and Systematic Review**

# II: Limitations for Search

**Search terms:**

1) intracerebral hemorrhage

2) intracerebral haemorrhage

3) sleep apnea

4) sleep apnoea

5) sleep disordered breathing

6) hemorrhagic stroke

7) stroke

8) sleep apnea screening

9) Apnea Hypopnea Index (AHI)

10) clinical settings

**Search Combination**

1) (1 or 2) and 8-10

2) (1 or 2) and (3 or 4)

3) (1 or 2) and 5

4) 5 and 6

5) 6 and (3 or 4)

6) 7 and (3 or 4 or 5)

Date/Time: No

Language: No

Document Type: No

Publication Status: No

# III: Resources and Number of Results

**Table S3** Search resources details and number of results

| Resource | Time Coverage | Search Interface | # of Hits |
|---|---|---|---|
| PubMed | 4.21.20 | PubMed.com | 224827 |
| Embase | 4.21.20 | Embase.com | 12,986 |
| Scopus | Until 4.21.20 | Scopus.com | 7,791 |
| ClinicalTrials.Gov | Until 4.21.2020 | ClinicalTrials.Gov | 172 |
| Forward and Backward citation tracking | Until 4.22.2020 | | 4 |
| **Results** | | | |
| Subtotal | | | 194346 |
| Adjusted* | | | 66,099 |
| Duplicates | | | 3,975 |
| Total | | | 38,456 |
| Mendeley | | | 8,800 |
| Full Text Screening | | | 200 |
| Data registry/RCT contacted | | | 30 |

**A maximum of 10,000 studies were imported per search result. The first 1,000 abstracts were screened for every search in Mendeley.*

# IV: Studies Excluded from Meta-Analysis

| Study | Prevalence of Intracerebral hemorrhage available | Author Contacted | Response from corresponding author | Other issues |
|---|---|---|---|---|
| **Aaraonson** [86,87] | Yes | Yes | No | Not available; Duplicative data; |
| **Arzt** [88] | No | Yes | No | - |

| | | | | |
|---|---|---|---|---|
| **Bochkarev [89]** | Yes | No | No | Questionnaire |
| **Bradley [90]** | No | Yes | No | - |
| **Brooks [91]** | No | Yes | No | - |
| **Brunner [92]** | Yes | Yes | Yes | AHI |
| **Camilo [14]** | Yes | Yes | Yes | Duplicate data |
| **Cao [93]** | No | Yes | No | - |
| **Castello-Branco [94]** | Yes | No | - | Questionnaire |
| **Colelli[95]** | Yes | No | No | - |
| **Dharmakulaseelan [96]** | No | No | No | |
| **Disler [97]** | No | Yes | No | - |
| **Dongmei [98]** | - | Yes | No | - |
| **Fournier [99]** | No | No | - | Abstract |
| **Harbinson [100]** | No | Yes | No | - |
| **Harbison [101]** | No | Yes | No | - |
| **Howook Kim [102]** | Yes | Yes | No | - |
| **Hsu [103]** | Yes | Yes | Yes | - |
| **Geer [104]** | No | No | No | Abstract; questionnaire |
| **Katzan [105]** | Yes | No | - | - |
| **Kendzerska [106]** | Yes | No | No | - |
| **Klobučníková [107]** | Yes | Yes | No | - |
| **Khot [108]** | No | - | - | |
| **Katzan [105]** | Yes | No | No | Questionnaire |
| **Kotzian [109–111]** | - | Yes | No | Not available |
| **Lin [112]** | Yes | Yes | No | - |
| **Mansour[113]** | No | No | - | Questionnaire |
| **Martynowicz [114]** | Yes | Yes | No | - |
| **Mohammad [115]** | No | No | - | Questionnaire |
| **Molano [116]** | No | No | - | Abstract; Questionnaire |

| | | | | |
|---|---|---|---|---|
| **Nasr [117]** | No | Yes | No | - |
| **Pajediene [118]** | No | Yes | Yes | - |
| **Parra [119]** | No | Yes | No | Duplicative data |
| **Patel [120]** | Yes | No | - | |
| **Ponsaing[121]** | Yes | - | - | |
| **Ryan [90]** | No | Yes | No | - |
| **Sahlin [72]** | Yes | - | - | Duplicative data |
| **Saletu [122]** | Yes | No | No | Not available |
| **Sandberg [123]** | Yes | Yes | No | Duplicative data; Not available |
| **Sarfo [124]** | - | - | - | |
| **Slonkova [125]** | Yes | Yes | | Not available |
| **Szücs [20]** | Yes | Yes | No | Device |
| **Terzoudi [126]** | No | No | No | - |
| **Wessendorf [127]** | No | Yes | No | - |
| **Wang [74]** | Yes | No | No | Not available |
| **Wen [128]** | Yes | No | - | Use snoring |

**SDB:** sleep disordered breathing; **AHI:** apnea-hypopnea index not available; **Questionnaire**: Used questionnaire to define SDB; **Snoring:** Used snoring to define SDB; **Not available:** information not available; **Duplicative data:** duplicative data could not be ruled out.

# V: Search Strategies

## A. *PubMed*

| | | |
|---|---|---|
| 1 | ((((intracerebral hemorrhage[Title/Abstract]) AND (intracerebral haemorrhage[Title/Abstract])) AND (Sleep apnea screening[Title/Abstract])) OR (apnea[Title/Abstract])) OR (clinical setting[Title/Abstract]) | 105,990 |
| 2 | (((intracerebral hemorrhage[Title/Abstract]) AND (intracerebral haemorrhage[Title/Abstract])) OR (sleep apnea[Title/Abstract])) OR (sleep apnoea[Title/Abstract]) | 36,649 |
| 3 | ((intracerebral hemorrhage[Title/Abstract]) AND (intracerebral haemorrhage[Title/Abstract])) OR (sleep disordered breathing[Title/Abstract]) | 6,797 |
| 4 | (sleep disordered breathing[Title/Abstract]) AND (hemorrhagic stroke[Title/Abstract]) | 2 |
| 5 | ((hemorrhagic stroke[Title/Abstract]) OR (sleep apnea[Title/Abstract])) OR (sleep apnoea[Title/Abstract]) | 41,309 |
| 6 | (((stroke[Title/Abstract]) AND (sleep apnea[Title/Abstract])) OR (sleep apnoea[Title/Abstract])) OR (sleep disordered breathing[Title/Abstract]) | 13,303 |
| Total | | 173764 |

***B. Scopus***

| | | |
|---|---|---|
| 1. | ((((intracerebral hemorrhage[Title/Abstract]) AND (intracerebrael hemorrhaege[Title/Abstract])) AND (Sleep apnea screening[Title/Abstract])) OR (apnea[Title/Abstract])) OR (clinical setting[Title/Abstract]) | 11,110 |
| 2. | (((intracerebral hemorrhage[Title/Abstract]) AND (intracerebral haemorrhaege[Title/Abstract])) OR (sleep apnea[Title/Abstract])) OR (sleep apnoea[Title/Abstract]) | 439 |
| 3. | ((intracerebral, hemorrhage[Title/Abstract]) AND(intracerebral haemorrhage[Title/Abstract])) OR (sleep disordered breathing[Title/Abstract]) | 391 |
| 4. | (sleep disordered breathing[Title/Abstract]) AND (hemorrhagic stroke[Title/Abstract]) | 16 |
| 5. | ((hemorrhagic stroke[Title/Abstract]) OR (sleep apnea[Title/Abstract])) OR (sleep apnoea[Title/Abstract]) | 663 |
| 6. | (((stroke[Title/Abstract]) AND (sleep apnea[Title/Abstract])) OR (sleep apnoea[Title/Abstract])) OR (sleep disordered breathing[Title/Abstract]) | 367 |
| Total | | 12,619 |

C. ***Embase***

| | | |
|---|---|---|
| 1 | ((((intracerebral hemorrhage[Title/Abstract]) AND (intracerebral hemorrhaege[Title/Abstract])) AND (Sleep apnea screening[Title/Abstract])) OR (apnea[Title/Abstract])) OR (clinical setting[Title/Abstract]) | 6,697 |
| 2 | (((intracerebral hemorrhage[Title/Abstract]) AND (intracerebral haemorrhaege[Title/Abstract])) OR (sleep apnea[Title/Abstract])) OR (sleep apnoea[Title/Abstract]) | 252 |
| 3 | ((intracerebral hemorrhage[Title/Abstract]) AND (intracerebral haemorrhage[Title/Abstract])) OR (sleep disordered breathing[Title/Abstract]) | 171 |
| 4 | (sleep disordered breathing[Title/Abstract]) AND (hemorrhagic stroke[Title/Abstract]) | 2 |
| 5 | ((hemorrhagic stroke[Title/Abstract]) OR (sleep apnea[Title/Abstract])) OR (sleep apnoea[Title/Abstract]) | 533 |
| 6 | (((stroke[Title/Abstract]) AND (sleep apnea[Title/Abstract])) OR (sleep apnoea[Title/Abstract])) OR (sleep disordered breathing[Title/Abstract]) | 211 |
| Total | | 7,791 |

## *E. ClinicalTrials.Gov*

( Cerebrovascular disease OR hemorrhagic stroke OR Intracerebral hemorrhage OR stroke OR cerebrovascular accident ) AND ( sleep apnea OR sleep disordered breathing )

## References


14. Camilo, M.R.; Fernandes, R.M.F.F.; Sander, H.H.; Nobre, F.; Santos-Pontelli, T.; Santos, A.C.d.; Araujo, D.B.d.; Leite, J.P.; Pontes-Neto, O.M.; dos Santos, A.C.; et al. Supine sleep and positional sleep apnea after acute ischemic stroke and intracerebral hemorrhage. *Clinics* **2012**, *67*, 1357–1360. https://doi.org/10.6061/clinics/2012(12)02.
20. Szücs, A.; Vitrai, J.J.; Janszky, J.J.; Migléczi, G.; Bódizs, R.; Halász, P.; Nagy, Z.Z.; Szucs, A.; Vitrai, J.J.; Janszky, J.J.; et al. Pathological sleep apnoea frequency remains permanent in ischaemic stroke and it is transient in haemorrhagic stroke: EBSCOhost. *Eur. Neurol*. **2002**, *47*, 15–19. https://doi.org/10.1159/000047941.
72. Sahlin, C.; Sandberg, O.; Gustafson, Y.; Bucht, G.; Carlberg, B.; Stenlund, H.; Franklin, K.A. Obstructive Sleep Apnea Is a Risk Factor for Death in Patients With Stroke. *Arch. Intern. Med.* **2008**, *168*, 297–301. https://doi.org/10.1001/archinternmed.2007.70.
74. Wang, Y.Y.; Wang, Y.Y.; Chen, J.; Yi, X.; Dong, S.; Cao, L. Stroke patterns, topography and etiology in patients with obstructive sleep apnea-hypopnea syndrome. *Int. J. Clin. Exp. Med.* **2017**, *10*, 7137–7143.
86. Aaronson, J.A.; Hofman, W.F.; van Bennekom, C.A.; van Bezeij, T.; Aardweg, J.G.v.D.; Groet, E.; Kylstra, W.A.; Schmand, B. Effects of Continuous Positive Airway Pressure on Cognitive and Functional Outcome of Stroke Patients with Obstructive Sleep Apnea: A Randomized Controlled Trial. *J. Clin. Sleep Med.* **2016**, *12*, 533–541, https://doi.org/10.5664/jcsm.5684.
87. Aaronson, J.A.; Nachtegaal, J.; van Bezeij, T.; Groet, E.; Hofman, W.F.; Aardweg, J.G.v.D.; van Bennekom, C.A. Can a Prediction Model Combining Self-Reported Symptoms, Sociodemographic and Clinical Features Serve as a Reliable First Screening Method for Sleep Apnea Syndrome in Patients With Stroke?. *Arch. Phys. Med. Rehabilitation* **2014**, *95*, 747–752, https://doi.org/10.1016/j.apmr.2013.12.011.
88. Arzt, M.; Young, T.; Peppard, P.E.; Finn, L.; Ryan, C.M.; Bayley, M.; Bradley, T.D. Dissociation of Obstructive Sleep Apnea From Hypersomnolence and Obesity in Patients With Stroke. *Stroke* **2010**, *41*, e129–34, https://doi.org/10.1161/strokeaha.109.566463.
89. Bochkarev, M. V. *et al.* Narusheniia sna i insul't: dannye issled0; Sleep Disorders and Stroke: Data of the Esse-Rf Study. *Zhurnal Nevrol. i psikhiatrii Im. S.S. Korsakova* 2019, *119*, 73–80. https://doi.org/10.17116/jnevro201911904273.
90. Ryan, C.M.; Bayley, M.; Green, R.; Murray, B.J.; Bradley, T.D. Influence of Continuous Positive Airway Pressure on Outcomes of Rehabilitation in Stroke Patients With Obstructive Sleep Apnea. *Stroke* **2011**, *42*, 1062–1067, https://doi.org/10.1161/strokeaha.110.597468.
91. Brooks, D.; Davis, L.; Vujovic-Zotovic, N.; Boulias, C.; Ismail, F.; Richardson, D.; Goldstein, R.S. Sleep-Disordered Breathing in Patients Enrolled in an Inpatient Stroke Rehabilitation Program. *Arch. Phys. Med. Rehabilitation* **2010**, *91*, 659–662, https://doi.org/10.1016/j.apmr.2009.12.019.
92. Brunner, H. Success and failure of mirtazapine as alternative treatment in elderly stroke patients with sleep apnea—a preliminary open trial. *Sleep Breath.* **2008**, *12*, 281–285, https://doi.org/10.1007/s11325-008-0177-7.
93. Cao, X.; Fan, C.; Bradley, T.D. An inpatient program for diagnosing and treating sleep apnea in patients with stroke. *Clin. Transl. Neurosci.* **2018**, *2*, https://doi.org/10.1177/2514183x18786843.

94. Castello-Branco, R.C.; Cerqueira-Silva, T.; Andrade, A.L.; Gonçalves, B.M.M.; Pereira, C.B.; Felix, I.F.; Santos, L.S.B.; Porto, L.M.; Marques, M.E.L.; Catto, M.B.; et al. Association Between Risk of Obstructive Sleep Apnea and Cerebrovascular Reactivity in Stroke Patients. *J. Am. Hear. Assoc.* **2020**, *9*, e015313, https://doi.org/10.1161/jaha.119.015313.
95. Colelli, D.; Black, S.; Masellis, M.; Lim, A.; Boulos, M. Feasibility of a home sleep apnea test in a cognitively impaired population. *Sleep Med.* **2019**, *64*, S47–S48, https://doi.org/10.1016/j.sleep.2019.11.131.
96. Dharmakulaseelan, L.; Kirolos, N.; Kamra, M.; Armesto-Heys, A.; Bouthillier, C.; Runions, S.; Linkewich, E.; Murray, B.J.; Boulos, M.I. Educating Stroke/TIA Patients about Obstructive Sleep Apnea after Stroke: A Randomized Feasibility Study. *J. Stroke Cerebrovasc. Dis.* **2019**, *28*, 104317, https://doi.org/10.1016/j.jstrokecerebrovasdis.2019.104317.
97. Bolu, O.; Nnadi, C.; Damisa, E.; Braka, F.; Siddique, A.; Archer, W.R.; Bammeke, P.; Banda, R.; Higgins, J.; Edukugo, A.; et al. Progress Toward Poliomyelitis Eradication — Nigeria, January–December 2017. *Mmwr-Morbidity Mortal. Wkly. Rep.* **2018**, *67*, 253–256, https://doi.org/10.15585/mmwr.mm6708a5.
98. Ye, D.; Chen, C.; Song, D.; Shen, M.; Liu, H.; Zhang, S.; Zhang, H.; Li, J.; Yu, W.; Wang, Q. Oropharyngeal Muscle Exercise Therapy Improves Signs and Symptoms of Post-stroke Moderate Obstructive Sleep Apnea Syndrome. *Front. Neurol.* **2018**, *9*, 912, https://doi.org/10.3389/fneur.2018.00912.
99. Fournier, L.; Tallavajhula, S.; Okpala, M.; Vahidy, F.; Sharrief, A. 1037 Prevalence of Obstructive Sleep Apnea in Intracerebral Hemorrhage, A Retrospective Study. *Sleep* **2018**, *41*, A385–A385, https://doi.org/10.1093/sleep/zsy061.1036.
100. Harbison, J.; Ford, G.; James, O.; Gibson, G. Sleep-disordered breathing following acute stroke. *Qjm: Int. J. Med.* **2002**, *95*, 741–747, https://doi.org/10.1093/qjmed/95.11.741.
101. Harbison, J.; Gibson, G. Snoring, sleep apnoea and stroke: chicken or scrambled egg?. *Qjm: Int. J. Med.* **2000**, *93*, 647–654, https://doi.org/10.1093/qjmed/93.10.647.
102. Kim, H.; Im, S.; Park, J.I.; Kim, Y.; Sohn, M.K.; Jee, S. Improvement of Cognitive Function after Continuous Positive Airway Pressure Treatment for Subacute Stroke Patients with Obstructive Sleep Apnea: A Randomized Controlled Trial. *Brain Sci.* **2019**, *9*, 252. https://doi.org/10.3390/brainsci9100252.
103. Hsu, C.-Y.; Vennelle, M.; Li, H.-Y.; Engleman, H.M.; Dennis, M.S.; Douglas, N.J. Sleep-disordered breathing after stroke: a randomised controlled trial of continuous positive airway pressure. *J. Neurol. Neurosurg. Psychiatry* **2006**, *77*, 1143–1149, https://doi.org/10.1136/jnnp.2005.086686.
104. Geer, J.H.; Leasure, A.C.; Vanent, K.N.; Sansing, L.H.; Woo, D.; Langefeld, C.; Yaggi, H.; Falcone, G.J.; Sheth, K.N. Abstract TP347: Obstructive Sleep Apnea Frequency in Intracerebral Hemorrhage. *Stroke* **2020**, *51*. https://doi.org/10.1161/str.51.suppl_1.tp347.
105. Katzan, I.L.; Thompson, N.R.; Walia, H.K.; Moul, D.E.; Foldvary-Schaefer, N. Sleep-related symptoms in patients with mild stroke. *J. Clin. Sleep Med.* **2020**, *16*, 55–64, https://doi.org/10.5664/jcsm.8122.
106. Kendzerska, T.; Wilton, K.; Bahar, R.; Ryan, C.M. Short- and long-term continuous positive airway pressure usage in the post-stroke population with obstructive sleep apnea. *Sleep Breath.* **2019**, *23*, 1233–1244, https://doi.org/10.1007/s11325-019-01811-9.
107. Klobučníková, K.; Šiarnik, P.; Čarnická, Z.; Kollár, B.; Turčáni, P. Causes of Excessive Daytime Sleepiness in Patients with Acute Stroke—A Polysomnographic Study. *J. Stroke Cerebrovasc. Dis.* **2016**, *25*, 83–86. https://doi.org/10.1016/j.jstrokecerebrovasdis.2015.08.038.
108. Khot, S.; Barnett, H.; Davis, A.; Siv, J.; Crane, D.; Kunze, A.; Lue, D.L.; Bunnell, A.; McCann, B.; Bombardier, C.; et al. Intensive Continuous Positive Airway Pressure Adherence Program During Stroke Rehabilitation. *Stroke* **2019**, *50*, 1895–1897, https://doi.org/10.1161/strokeaha.119.024795.
109. Kotzian, S.T.; Stanek, J.K.; Pinter, M.M.; Grossmann, W.; Saletu, M.T. Subjective Evaluation of Sleep Apnea Is Not Sufficient in Stroke Rehabilitation. *Top. Stroke Rehabilitation* **2012**, *19*, 45–53, https://doi.org/10.1310/tsr1901-45.
110. Kotzian, S.T.; Schwarzinger, A.; Haider, S.; Saletu, B.; Spatt, J.; Saletu, M.T. Home polygraphic recording with telemedicine monitoring for diagnosis and treatment of sleep apnoea in stroke (HOPES Study): study protocol for a single-blind, randomised controlled trial. *BMJ Open* **2018**, *8*, e018847, https://doi.org/10.1136/bmjopen-2017-018847.
111. Kotzian, S.T.; Saletu, M.T.; Schwarzinger, A.; Haider, S.; Spatt, J.; Kranz, G.; Saletu, B. Proactive telemedicine monitoring of sleep apnea treatment improves adherence in people with stroke– a randomized controlled trial (HOPES study). *Sleep Med.* **2019**, *64*, 48–55, https://doi.org/10.1016/j.sleep.2019.06.004.

112. Lin, S.H.; Branson, C.; Park, L.; Leung, J.; Doshi, N.; Auerbach, S.H. Oximetry as an Accurate Tool for Identifying Moderate to Severe Sleep Apnea in Patients With Acute Stroke. *J. Clin. Sleep Med.* **2018**, *14*, 2065–2073, https://doi.org/10.5664/jcsm.7538.
113. Mansour, A.H.; Ayad, M.; El-Khayat, N.; El Sadek, A.; Alloush, T.K. Post-stroke sleep disorders in Egyptian patients by using simply administered questionnaires: a study from Ain Shams University. *Egypt. J. Neurol. Psychiatry Neurosurg.* **2020**, *56*, 1–6, https://doi.org/10.1186/s41983-020-0148-x.
114. Martynowicz, H.; Jodkowska, A.; Skomro, R.; Gać, P.; Brylka, A.; Bladowski, M.; Wojakowska, A.; Mazur, G.; Poręba, R. The estimation of excessive daytime sleepiness in post-stroke patients - a polysomnographic study. *Respir. Physiol. Neurobiol.* **2019**, *267*, 1–5, https://doi.org/10.1016/j.resp.2019.05.013.
115. Mohammad, Y.; Almutlaq, A.; Al-Ruwaita, A.; Aldrees, A.; Alsubaie, A.; Al-Hussain, F. Stroke during sleep and obstructive sleep apnea: there is a link. *Neurol. Sci.* **2019**, *40*, 1001–1005, https://doi.org/10.1007/s10072-019-03753-2.
116. Molano, J.R.V.; Koch, S.; Langefeld, C.; Woo, D. Abstract TP209: Time Of Stroke Onset Is Not Associated With Risk Of Obstructive Sleep Apnea In Intracerebral Hemorrhage.. *Stroke* **2013**, *44.* https://doi.org/10.1161/str.44.suppl_1.atp209.
117. Nasr-Wyler, A.; Bouillanne, O.; Lalhou, A.; Goldenberg, F.; Bissery, A.; Piette, F. [Sleep apnea syndrome and stroke in the elderly population].. **1999**, *155*, 1057–62.
118. Pajediene, E.; Pajeda, A.; Urnieziute, G.; Paulekas, E.; Liesiene, V.; Bileviciute-Ljungar, I.; Jurkeviciene, G.; Rastenyte, D.; Petrikonis, K. Subjective and objective features of sleep disorders in patients with acute ischemic or haemorrhagic stroke: It is not only sleep apnoea which is important. **2019**, *136*, 109512, https://doi.org/10.1016/j.mehy.2019.109512.
119. Parra, O.; Arboix, A. Stroke and sleep-disordered breathing: A relationship under construction. *World J. Clin. Cases* **2016**, *4*, 33–7, https://doi.org/10.12998/wjcc.v4.i2.33.
120. Patel, N.; Raissi, A.; Elias, S.; Kamra, M.; Kendzerska, T.; Murray, B.J.; Boulos, M.I. A Modified Definition for Obstructive Sleep Apnea in Home Sleep Apnea Testing after Stroke or Transient Ischemic Attack. *J. Stroke Cerebrovasc. Dis.* **2018**, *27*, 1524–1532, https://doi.org/10.1016/j.jstrokecerebrovasdis.2017.12.052.
121. Ponsaing, L.B.; Iversen, H.K.; Jennum, P. Polysomnographic indicators of mortality in stroke patients. *Sleep Breath.* **2016**, *21*, 235–242, https://doi.org/10.1007/s11325-016-1387-z.
122. Saletu, M.T.; Kotzian, S.T.; Schwarzinger, A.; Haider, S.; Spatt, J.; Saletu, B. Home Sleep Apnea Testing is a Feasible and Accurate Method to Diagnose Obstructive Sleep Apnea in Stroke Patients During In-Hospital Rehabilitation. *J. Clin. Sleep Med.* **2018**, *14*, 1495–1501, https://doi.org/10.5664/jcsm.7322.
123. Sandberg, O.; Franklin, K.; Bucht, G.; Eriksson, S.; Gustafson, Y. Nasal continuous positive airway pressure in stroke patients with sleep apnoea: a randomized treatment study. *Eur. Respir. J.* **2001**, *18*, 630–634, https://doi.org/10.1183/09031936.01.00070301.
124. Sarfo, F.S.; Jenkins, C.; Mensah, N.A.; Saulson, R.; Sarfo-Kantanka, O.; Singh, A.; Nichols, M.; Qanungo, S.; Ovbiagele, B. Prevalence and Predictors of Sleep Apnea Risk among Ghanaian Stroke Survivors. **2017**, *26*, 1602–1608, https://doi.org/10.1016/j.jstrokecerebrovasdis.2017.02.027.
125. Slonkova, J.; Bar, M.; Nilius, P.; Berankova, D.; Salounova, D.; Sonka, K. Spontaneous improvement in both obstructive sleep apnea and cognitive impairment after stroke. *Sleep Med.* **2017**, *32*, 137–142, https://doi.org/10.1016/j.sleep.2016.11.024.
126. Terzoudi, A.; Vorvolakos, T.; Heliopoulos, I.; Livaditis, M.; Vadikolias, K.; Piperidou, H. Sleep Architecture in Stroke and Relation to Outcome. *Eur. Neurol.* **2008**, *61*, 16–22, https://doi.org/10.1159/000165344.
127. Wessendorf, T.E.; Teschler, H.; Wang, Y.-M.; Konietzko, N.; Thilmann, A.F. Sleep-disordered breathing among patients with first-ever stroke. *J. Neurol.* **2000**, *247*, 41–47, https://doi.org/10.1007/pl00007787.
128. Wen, Y.; Pi, F.-H.; Guo, P.; Dong, W.-Y.; Xie, Y.-Q.; Wang, X.-Y.; Xia, F.-F.; Pang, S.-J.; Wu, Y.-C.; Wang, Y.-Y.; et al. Sleep duration, daytime napping, markers of obstructive sleep apnea and stroke in a population of southern China. *Sci. Rep.* **2016**, *6*, 34689, https://doi.org/10.1038/srep34689.


**Disclaimer/Publisher's Note:** The statements, opinions and data contained in all publications are solely those of the individual author(s) and contributor(s) and not of MDPI and/or the editor(s). MDPI and/or the editor(s) disclaim responsibility for any injury to people or property resulting from any ideas, methods, instructions or products referred to in the content.
