# Supplementary material for: Global Prevalence of Sleep-Disordered Breathing in Intracerebral Hemorrhage Survivors: A Meta-Analysis and Systematic Review
Source: Neurol Int. 2026 Jan 20;18(1):19. doi: 10.3390/neurolint18010019 (PMC12845411; doi:10.3390/neurolint18010019)
Supplement: Supplementary file 1 [file neurolint-18-00019-s001.zip › supplementary material S3- Percentage of Patients with SDB by subgroup.pdf]

Table S4. Percentage of Patients with SDB by subgroups AHI>5

Variable	Group	% SDB (95% CI)	I^2	I^2 p-value	I^2 Between Groups p-value	ES p-value
**Location**	Rehab Unit	88% (83–92)	—	—	0.26	<0.001
	Cohort	84% (73–91)	—	—		<0.001
	Inpatient	82% (63–96)	68.8%	0.01		<0.001
	Home	67% (39–86)	—	—		<0.001
	Sleep Lab	83% (67–95)	—	—		<0.001
	Overall	85% (77–91)	54.7%	0.02		<0.001
**Timing 1**	30D to 3Months	88% (83–93)	—	—	0.29	<0.001
	<30D	85% (78–93)	49.5%	0.06	—	<0.001
	>3Months	67% (39–86)	—	—	—	<0.001
	Overall	85% (80–91)	47.5%	0.05	—	<0.001
**Timing 2**	30D	79% (63–95)	—	—	0.73	<0.001
	<7D	82% (67–96)	67.9%	0.02	—	<0.001
	7-30D	86% (78–93)	—	—	—	<0.001
	Overall	85% (80–91)	47.5%	0.05	—	<0.001
**Hypopnea Criteria**	b	86% (68–98)	—	—	0.37	<0.001
	a	86% (79–92)	20.5%	0.28		<0.001
	3	67% (39–86)	—	—		<0.001
	Overall	85% (77–91)	54.7%	0.02		<0.001
**Hypopnea Type**	2	89% (82–95)	54.1%	0.07	0.12	<0.001
	7	50% (19–81)	—	—		0.003
	3	67% (39–86)	—	—		<0.001
	1	87% (71–98)	—	—		<0.001
	5	78% (61–89)	—	—		<0.001
	Overall	85% (77–91)	54.7%	0.02		<0.001
**Device Type**	3	86% (77–94)	69.5%	0.01	0.61	<0.001
	1	81% (70–91)	0%	0.50		<0.001
	2	78% (45–94)	—	—		<0.001
	Overall	85% (77–91)	54.7%	0.02		<0.001
**Country**	Australia	61% (37–84)	—	—	0.01	<0.001
	USA	86% (75–95)	—	—		<0.001
	India	90% (70–97)	—	—		<0.001
	Japan	91% (87–94)	—	—		<0.001
	Brazil	78% (61–89)	—	—		<0.001
	Russia	78% (45–94)	—	—		<0.001
	Chi—	69% (42–87)	—	—		<0.001
	Overall	85% (77–91)	54.7%	0.02		<0.001
**Continent**	Europe	78% (45–94)	—	—	0.07	<0.001
	North America	86% (75–95)	—	—		<0.001
	Australia	61% (37–84)	—	—		<0.001
	Asia	90% (83–95)	51.7%	0.10		<0.001
	South America	78% (61–89)	—	—		<0.001
	Overall	85% (77–91)	54.7%	0.02		<0.001
**Study Type**	Retrospective	88% (83–92)	—	—	0.09	<0.001
	Prospective	82% (72–91)	57.7%	0.02		<0.001
	Overall	85% (77–91)	54.7%	0.02		<0.001

CI: confidence interval. The p-values indicate statistical significance of the subgroups of percentage of patients with SDB obtained with a random ef**fects model. Patient Location: Home, Inpatient (stroke unit or hospital), Cohort (patients at home), sleep Lab; AASM Device: 1-4; Full vs. Limited: Full channel device (AASM 1-2), Limited channel device (AASM 3-4);**); **Hypopnea Criteria** A (3 %), B (4 %)**; Hypopnea Type AASM:** 1-7

Table S5. Percentage of Patients with SDB by subgroups AHI>10

Variable	Group	% SDB (95% CI)	I^2	I^2p-value	I^2 Between-Group p-value	ES p-value
**Location**	Rehab Unit	68% (60–74)	0%	0.41		<0.001
	Cohort	48% (35–60)	—	—		<0.001
	Inpatient	70% (53–85)	15%	0.32		<0.001
	Home	58% (32–81)	—	—		<0.001
	Sleep Lab	58% (32–81)	—	—		<0.001
	—	63% (55–70)	26%	0.19	0.08	<0.001
**Timing 1**	30D to 3Months	69% (60–79)	—	—	0.51	<0.001
	<30D	62% (48–76)	61.4%	0.02	—	<0.001
	>3Months	58% (39–78)	—	—	—	<0.001
	Overall	64% (56–72)	47.4%	0.03	—	<0.001

**Variable**	**Group**	**% SDB (95% CI)**	**I²**	**I²p-value**	**I² Between-Group p-value**	**ES p-value**
**Timing 2**	>30D	67% (61–74)	0%	0.51	0.02	<0.001
	<7D	71% (48–93)	—	—	—	<0.001
	7-30D	51% (41–61)	0%	0.54	—	<0.001
	Overall	64% (56–72)	47.4%	0.03	—	<0.001
**Hypopnea Criteria**	b	47% (34–60)	—	—		<0.001
	a	68% (61–74)	0%	0.69		<0.001
	3	65% (44–84)	—	—		<0.001
	Overall	63% (55–70)	26%	0.19	0.02	<0.001
**Hypopnea Type**	2	60% (44–75)	62%	0.05		<0.001
	7	50% (19–81)	—	—		<0.001
	3	74% (53–91)	—	—		<0.001
	4	83% (55–95)	—	—		<0.001
	1	59% (40–78)	—	—		<0.001
	5	63% (45–77)	—	—		<0.001
	Overall	63% (55–70)	26%	0.19	0.59	<0.001
**Device Type**	3	62% (49–74)	57%	0.04		<0.001
	4	53% (28–77)	—	—		<0.001
	1	68% (54–80)	0%	0.56		<0.001
	Overall	63% (55–70)	26%	0.19	0.58	<0.001
**Country**	Ca—da	75% (51–93)	—	—		<0.001
	Australia	56% (31–79)	—	—		<0.001
	USA	50% (38–62)	—	—		<0.001
	France	50% (24–76)	—	—		<0.001
	Japan	67% (60–74)	—	—		<0.001
	Spain	90% (60–98)	—	—		<0.001
	Brazil	63% (45–77)	—	—		<0.001
	Sweden	60% (36–80)	—	—		<0.001
	Overall	63% (55–70)	26%	0.19	0.10	<0.001
**Continent**	Europe	67% (43–88)	—	—		<0.001
	North America	60% (44–75)	35%	0.19		<0.001
	Australia	56% (31–79)	—	—		<0.001
	Asia	67% (60–74)	—	—		<0.001
	South America	63% (45–77)	—	—		<0.001
	Overall	63% (55–70)	26%	0.19	0.77	<0.001
**Study Type**	Retrospective	67% (60–74)	—	—		<0.001
	Prospective	61% (51–71)	25%	0.22		<0.001
	Overall	63% (55–70)	26%	0.19	0.30	<0.001

CI: confidence interval. The p-values indicate statistical significance of the subgroups of percentage of patients with SDB obtained with a random effects model. **Patient Location:** Home, Inpatient (stroke unit or hospital), Cohort (patients at home), sleep Lab; **AASM Device:** 1-4; **Full vs. Limited:** Full channel device (AASM 1-2), Limited channel device (AASM 3-4); **); Hypopnea Criteria A (3 %), B (4 %); Hypopnea Type AASM: 1-7**

Table S6. Percentage of Patients with SDB by subgroups AHI>15

**Variable**	**Group**	**% SDB (95% CI)**	**I² (%)**	**I²p-value**	**I² Between-Groups p-value**	**ES p-value**
**Location**	Rehab Unit	47% (39–54)	—	—		<0.001
	Cohort	35% (25–48)	—	—		<0.001
	Inpatient	64% (55–72)	0.0	0.78		<0.001
	Home	42% (19–68)	—	—		<0.001
	Sleep Lab	48% (31–66)	—	—		<0.001
	Overall	50% (42–57)	40.1	0.08	0.00	<0.001
**Timing 1**	30D to 3Months	48% (41–55)	—	—	0.86	<0.001
	<30D	50% (38–62)	63.7%	0.01	—	<0.001
	>3Months	42% (19–68)	—	—	—	<0.001
	Overall	49% (42–57)	44.8%	0.05	—	<0.001
**Timing 2**	>30D	47% (41–54)	0%	0.94	<0.001	<0.001
	<7D	63% (55–71)	—	—	—	<0.001
	7-30D	38% (29–48)	0%	0.85	—	<0.001
	Overall	49% (42–57)	44.8%	0.05	—	<0.001
**Hypopnea Criteria**	b	50% (30–70)	77.9	0.00		<0.001
	a	49% (42–55)	0.0	0.83		<0.001
	3	42% (19–68)	—	—		<0.001
	Overall	50% (42–57)	40.1	0.08	0.90	<0.001
**Hypopnea Type**	2	50% (38–61)	65.7	0.01		<0.001
	7	50% (19–81)	—	—		<0.001
	3	42% (19–68)	—	—		<0.001
	1	43% (26–60)	—	—		<0.001
	5	59% (42–74)	—	—		<0.001
	Overall	50% (42–57)	40.1	0.08	0.73	<0.001
**Device Type**	3	48% (37–58)	60.9	0.02		<0.001
	1	54% (41–66)	0.0	0.80		<0.001
	Overall	50% (42–57)	40.1	0.08	0.47	<0.001
**Country**	Netherlands	45% (21–72)	—	—		<0.001
	Australia	44% (21–69)	—	—		<0.001
	USA	35% (23–48)	—	—		<0.001
	India	45% (26–66)	—	—		<0.001
	Japan	54% (48–60)	—	—		<0.001
	Brazil	59% (42–74)	—	—		<0.001
	Sweden	40% (20–64)	—	—		<0.001
	China	54% (29–77)	—	—		<0.001
	Overall	50% (42–57)	40.1	0.08	0.27	<0.001
**Continent**	Europe	42% (23–62)	—	—		<0.001
	North America	35% (23–48)	—	—		<0.001
	Australia	44% (21–69)	—	—		<0.001
	Asia	54% (42–65)	62.7	0.05		<0.001
	South America	59% (42–74)	—	—		<0.001
	Overall	50% (42–57)	40.1	0.08	0.17	<0.001
**Study Type**	Retrospective	47% (40–55)	—	—		<0.001
	Prospective	50% (40–60)	49.3	0.05		<0.001
	Overall	50% (42–57)	40.1	0.08	0.69	<0.001

CI: confidence interval. The p-values indicate statistical significance of the subgroups of percentage of patients with SDB obtained with a random effects model. **Patient Location:** Home, Inpatient (stroke unit or hospital), Cohort (patients at home), sleep Lab; **AASM Device:** 1-4; **Full vs. Limited:** Full channel device (AASM 1-2), Limited channel device (AASM 3-4); **); Hypopnea Criteria A (3 %), B (4 %); Hypopnea Type AASM: 1-7**

Table S7. Percentage of Patients with SDB by subgroups AHI>20

Variable	Group	% SDB (95% CI)	I² (%)	I² p-value	Between-Groups p-value	ES p-value
**Location**	Rehab Unit	34% (27–41)				<0.001
	Inpatient	47% (31–64)				<0.001
	Home	42% (19–68)				<0.001
	Overall	36% (29–44)	6.2	0.36	0.25	<0.001
**Timing 1**	30D to 3Months	34% (27–41)	—	—	0.29	<0.001
	<30D	47% (31–63)	—	—	—	<0.001
	>3Months	42% (19–68)	—	—	—	<0.001
	Overall	37% (30–44)	4.1%	0.37	—	<0.001
**Timing 2**	>30D	34% (27–41)	—	—	0.14	<0.001
	<7D	47% (31–63)	—	—	—	<0.001
	Overall	36% (29–44)	6.2	0.36	0.12	<0.001
**Hypopnea Criteria**	b	33% (10–70)				0.02
	a	36% (29–43)				<0.001
	3	42% (19–68)				<0.001
	Overall	36% (29–44)	6.2	0.36	0.91	<0.001
**Hypopnea Type**	2	34% (27–41)				<0.001
	7	33% (10–70)				0.02
	3	42% (19–68)				<0.001
	5	50% (34–66)				<0.001
	Overall	36% (29–44)	6.2	0.36	0.36	<0.001
**Device Type**	3	33% (26–41)				<0.001
	1	50% (34–66)				<0.001
	Overall	36% (29–44)	6.2	0.36	0.09	<0.001
**Country**	Australia	39% (16–64)				<0.001
	Japan	34% (27–41)				<0.001
	Brazil	50% (34–66)				<0.001
	Overall	36% (29–44)	6.2	0.36	0.21	<0.001
**Continent**	Australia	39% (16–64)				<0.001
	Asia	34% (27–41)				<0.001
	South America	50% (34–66)				<0.001
	Overall	36% (29–44)	6.2	0.36	0.21	<0.001
**Study Type**	Retrospective	34% (27–41)				<0.001
	Prospective	46% (31–61)				<0.001
	Overall	36% (29–44)	6.2	0.36	0.11	<0.001

CI: confidence interval. The p-values indicate statistical significance of the subgroups of percentage of patients with SDB obtained with a random effects model. **Patient Location:** Home, Inpatient (stroke unit or hospital), Cohort (patients at home), sleep Lab; **AASM Device:** 1-4; **Full vs. Limited:** Full channel device (AASM 1-2), Limited channel device (AASM 3-4); **Hypopnea Criteria** A (3 %), B (4 %)**; Hypopnea Type AASM:** 1-7

Table S8. Percentage of Patients with SDB by subgroups AHI>30

Variable	Group	% SDB (95% CI)	I² (%)	I² p-value	I² Between-Groups p-value	ES p-value
**Location**	Rehab Unit	15% (11–22)	—	—		<0.001
	Cohort	19% (11–31)	—	—		<0.001
	Inpatient	29% (22–38)	0.0	0.89		<0.001
	Home	8% (1–35)	—	—		0.15
	Sleep Lab	20% (8–42)	—	—		<0.001
	Overall	21% (15–28)	43.2	0.09	0.02	<0.001
**Timing 1**	30D to 3Months	15% (11–22)	—	—	0.01	<0.001
	<30D	26% (20–31)	0%	0.56	—	<0.001
	>3Months	8% (1–35)	—	—	—	0.30
	Overall	21% (15–27)	44.9%	0.08	—	<0.001
**Timing 2**	>30D	14% (9–20)	—	—	<0.001	<0.001
	<7D	30% (23–38)	0%	0.92	—	<0.001
	7-30D	20% (11–28)	—	—	—	<0.001
	Overall	21% (15–27)	44.9%	0.08	—	<0.001
**Hypopnea Criteria**	b	25% (17–33)	—	—		<0.001
	a	21% (12–32)	47.5	0.13		<0.001
	3	8% (1–35)	—	—		0.15
	Overall	21% (15–28)	43.2	0.09	0.40	<0.001
**Hypopnea Type**	2	21% (13–31)	—	—		<0.001
	7	33% (10–70)	—	—		0.02
	3	21% (5–41)	—	—		<0.001
	1	20% (8–42)	—	—		<0.001
	5	28% (16–45)	—	—		<0.001
	Overall	21% (15–28)	43.2	0.09	0.88	<0.001
**Device Type**	3	21% (13–30)	55.8	0.05		<0.001
	1	25% (14–38)	—	—		<0.001
	Overall	21% (15–28)	43.2	0.09	0.63	<0.001
**Country**	Australia	15% (1–37)	—	—		0.02
	USA	19% (11–31)	—	—		<0.001
	India	20% (8–42)	—	—		<0.001
	Japan	20% (16–25)	—	—		<0.001
	Spain	40% (17–69)	—	—		<0.001
	Brazil	28% (16–45)	—	—		<0.001
	Overall	21% (15–28)	43.2	0.09	0.67	<0.001
**Continent**	Europe	40% (17–69)	—	—		<0.001
	North America	19% (11–31)	—	—		<0.001
	Australia	15% (1–37)	—	—		0.02
	Asia	21% (11–33)	—	—		<0.001
	South America	28% (16–45)	—	—		<0.001
	Overall	21% (15–28)	43.2	0.09	0.59	<0.001
**Study Type**	Retrospective	15% (11–22)	—	—		<0.001
	Prospective	24% (19–30)	0.0	0.45		<0.001
	Overall	21% (15–28)	43.2	0.09	0.01	<0.001

CI: confidence interval. The p-values indicate statistical significance of the subgroups of percentage of patients with SDB obtained with a random effects model. **Patient Location:** Home, Inpatient (stroke unit or hospital), Cohort (patients at home), sleep Lab; **AASM Device:** 1-4; **Full vs. Limited:** Full channel device (AASM 1-2), Limited channel device (AASM 3-4); **Hypopnea Criteria A (3 %), B (4 %); Hypopnea Type AASM: 1-7**

Table S9. Percentage of Patients with SDB by subgroups AHI>40

Variable	Group	% SDB (95% CI)	I^2 (%)	I^2 p-value	I^2 Between-Groups p-value	ES p-value
**Location**	Rehab Unit	9% (5–14)	—	—		<0.001
	Inpatient	20% (7–35)	—	—		<0.001
	Overall	13% (3–28)	61.4	0.08	0.03	<0.001
**Timing 1**	30D to 3Months	9% (5–14)	—	—	0.08	<0.001
	<30D	20% (8–33)	—	—	—	<0.001
	Overall	13% (3–23)	—	—	—	0.01
**Timing 2**	>30D	9% (5–14)	—	—	0.08	<0.001
	<7D	20% (8–33)	—	—	—	<0.001
	Overall	13% (3–23)	—	—	—	0.01
**Hypopnea Criteria**	b	33% (10–70)	—	—		0.02
	a	10% (6–14)	—	—		<0.001
	Overall	13% (3–28)	61.4	0.08	0.12	<0.001
**Hypopnea Type**	2	9% (5–14)	—	—		<0.001
	7	33% (10–70)	—	—		0.02
	5	19% (9–35)	—	—		<0.001
	Overall	13% (3–28)	61.4	0.08	0.08	<0.001
**Device Type**	3	6% (2–12)	—	—		<0.001
	1	19% (9–35)	—	—		<0.001
	Overall	13% (3–28)	61.4	0.08	0.13	<0.001
**Country**	Australia	33% (10–70)	—	—		0.02
	Japan	9% (5–14)	—	—		<0.001
	Brazil	19% (9–35)	—	—		<0.001
	Overall	13% (3–28)	61.4	0.08	0.08	<0.001
**Continent**	Australia	33% (10–70)	—	—		0.02
	Asia	9% (5–14)	—	—		<0.001
	South America	19% (9–35)	—	—		<0.001
	Overall	13% (3–28)	61.4	0.08	0.08	<0.001
**Study Type**	Retrospective	9% (5–14)	—	—		<0.001
	Prospective	20% (7–35)	—	—		<0.001
	Overall	13% (3–28)	61.4	0.08	0.03	<0.001

CI: confidence interval. The p-values indicate statistical significance of the subgroups of percentage of patients with SDB obtained with a random effects model. **Patient Location:** Home, Inpatient (stroke unit or hospital), Cohort (patients at home), sleep Lab; **AASM Device:** 1-4; **Full vs. Limited:** Full channel device (AASM 1-2), Limited channel device (AASM 3-4); **Hypopnea Criteria A** (3 %), **B** (4 %); **Hypopnea Type AASM:** 1-7
