# Supplementary material for: Global Prevalence of Sleep-Disordered Breathing in Intracerebral Hemorrhage Survivors: A Meta-Analysis and Systematic Review
Source: Neurol Int. 2026 Jan 20;18(1):19. doi: 10.3390/neurolint18010019 (PMC12845411; doi:10.3390/neurolint18010019)
Supplement: Supplementary file 1 [file neurolint-18-00019-s001.zip › Supplementary Material S4- Risk of Bias Summary.pdf]

Figure S1.

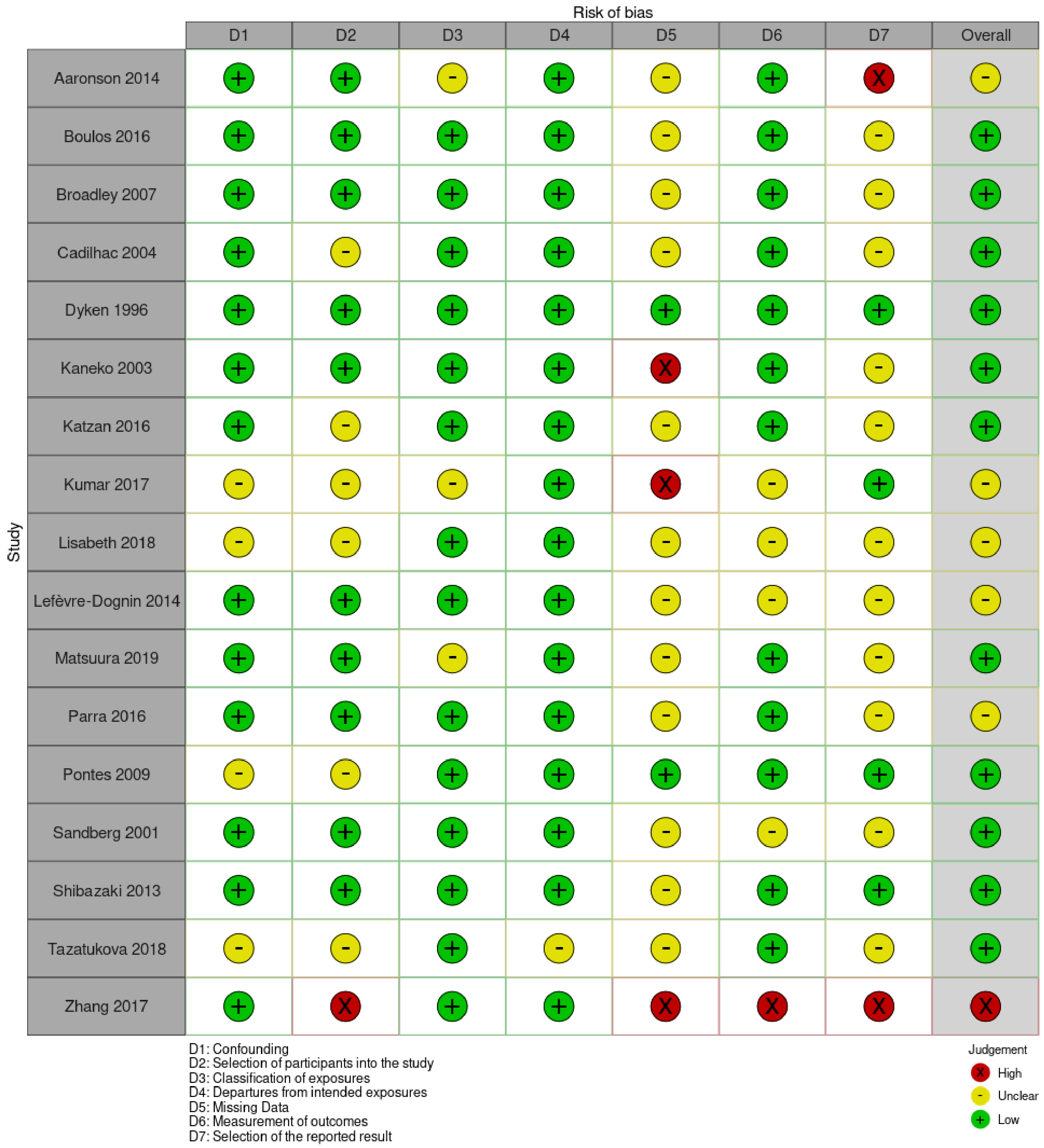

Study-level Risk of Bias Assessment using the ROBINS-I framework. Each domain is represented with color-coded judgments: green for low risk, yellow for unclear or some concerns, and red for high risk of bias. D1: Confounding; D2: Selection of participants; D3: Classification of exposures; D4: Departures from intended exposures; D5: Missing data; D6: Measurement of outcomes; D7: Selection of reported results.

Figure S2.

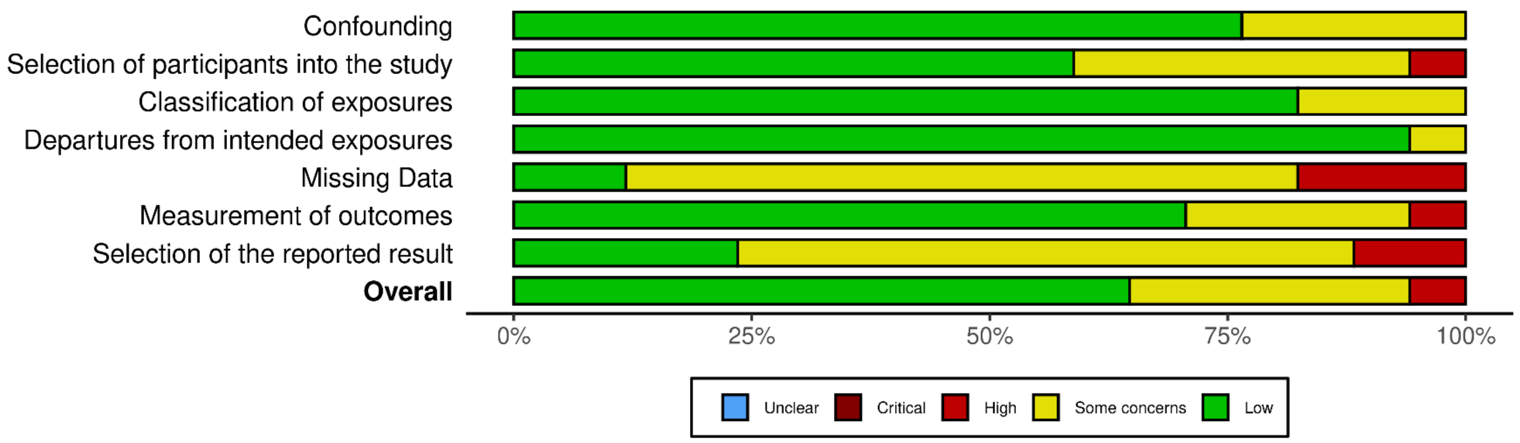

Summary of Risk of Bias across included studies. The proportion of studies rated as low risk (green), with some concerns (yellow), or high risk (red) is shown for each ROBINS-I domain and overall assessment.

Table S10. Summary of The Risk of Bias Assessment

	Confounding	Selection of participants into the study	Classification of exposures	Departures from intended exposures	Missing Data	Measurement of outcomes	Selection of the reported result	Overall Bias
**Aaronson 2014**	*	*	**	*	**	*	***	**
**Boulous 2016**	*	*	*	*	**	*	**	*
**Broadley 2007**	*	*	*	*	**	*	**	*
**Cadilhac 2004**	*	**	*	*	**	*	**	*
**Dyken 1996**	*	*	*	*	*	*	*	*
**Kaneko 2003**	*	*	*	*	***	*	**	*
**Katzan 2016**	*	**	*	*	**	*	**	*
**Kumar 2017**	**	**	**	*	***	**	*	**
**Lisabeth 2018**	**	**	*	*	**	**	**	**
**Lefèvre-Dognin 2014**	*	*	*	*	**	**	**	**
**Matsuura 2019**	*	*	**	*	**	*	**	*
**Parra 2016**	*	*	*	*	**	*	**	**
**Pontes 2009**	**	**	*	*	*	*	*	*
**Sandberg 2001**	*	*	*	*	**	**	**	*
**Shibazaki 2013**	*	*	*	*	**	*	*	*
**Tazatukova 2018**	**	**	*	**	**	*	**	*
**Zhang 2017**	*	***	*	*	***	***	***	***

* Low, ** Unknown, and ⁂ High risk of bias.

Table S11. Egger's Bias Test for Prevalence and Mean AHI

AHI Threshold	Beta Coef	SE of Beta	z-value	p-value	Evidence of Bias
**AHI > 5**	-1.71	0.984	-1.74	0.0818	Suggestive, borderline
**AHI > 10**	-1.07	1.120	-0.96	0.3391	No evidence
**AHI > 15**	0.77	1.089	0.71	0.4784	No evidence
**AHI > 20**	0.42	1.154	0.37	0.7132	No evidence
**AHI > 30**	0.17	0.921	0.18	0.8561	No evidence
**AHI > 40**	3.24	1.465	2.21	0.0271	Significant small-study bias
**Mean AHI**	1.61	1.264	1.27	0.2030	No evidence

Egger's test for small-study effects was performed across each apnea-hypopnea index (AHI) severity threshold to assess potential publication bias. For AHI > 5, there was a borderline signal of small-study effects (β = -1.71, SE = 0.98, p = 0.0818), suggesting possible bias at lower severity thresholds. No significant small-study effects were detected for AHI > 10 (β = -1.07, p = 0.3391), AHI > 15 (β = 0.77, p = 0.4784), AHI > 20 (β = 0.42, p = 0.7132), or AHI > 30 (β = 0.17, p = 0.8561). However, at the highest severity threshold (AHI > 40), Egger's test indicated statistically significant small-study effects (β = 3.24, p = 0.0271), suggesting potential bias in reporting or selective publication among studies evaluating severe sleep-disordered breathing in this population.

Table S12: Egger's Bias Test for RR and OR of OSA and CSA

Population	Measure	Pooled Estimate	95% Confidence Interval	Heterogeneity (I^2)	p for Heterogeneity	Egger's Test p-value
**ICH Survivors with SDB**	Risk Ratio	7.29	2.70 to 19.89	60.3%	0.053	0.8700
	Odds Ratio	53.08	8.44 to 333.99	84.1%	<0.001	0.6300
**ICH Survivors**	Risk Ratio	7.44	2.79 to 19.81	56.1%	0.077	0.8654
	Odds Ratio	53.08	8.44 to 334.00	84.1%	<0.001	0.7465

Egger's test showed no significant small-study effects in either analysis
